# A dynamic scapping workflow for RTK domains: computational modeling of natural products as dual modulators of EGFR and VEGFR signaling in breast cancer

**DOI:** 10.1007/s11030-025-11263-x

**Published:** 2025-07-10

**Authors:** Vincent A. Obakachi, Krishna K. Govender, Penny P. Govender

**Affiliations:** Department of Chemical Sciences, Doornfontein Campus, University of Johannesburg, P.O. Box 17011, Johannesburg, 2028 South Africa

**Keywords:** Breast cancer, RTKs, Natural products, Molecular dynamics, MM/GBSA, Dual modulators

## Abstract

Breast cancer, a major global health challenge, is driven by aberrant receptor tyrosine kinase (RTK) signaling via epidermal growth factor receptor (EGFR) and vascular endothelial growth factor receptor (VEGFR). This study employs a "Dynamic Scapping" workflow, integrating molecular docking, 500 ns molecular dynamics (MD) simulations, and MM/GBSA binding free energy calculations to identify natural products with potential for dual binding to EGFR (PDB: 1M17) and VEGFR (PDB: 3VHE). From ~ 20,000 natural products, virtual screening shortlisted 13 EGFR and 12 VEGFR hits, with Digitonin, Cyclamin, Vicenin-2, Glucosylorientin, and Nicotiflorin selected for EGFR, and Quercetagetin, Silychristin, Quercetin, Scutellarein, and Isorhamnetin for VEGFR, alongside references (Erlotinib, Pyrrolopyrimidine). MD simulations, conducted as single trajectories per system, revealed stable complexes (RMSD: 1.73–2.92 Å), with Digitonin, Cyclamin, and Silychristin showing binding energies (ΔG_bind_: − 84.29, − 81.47, − 63.33 kcal/mol) compared to references (Erlotinib: − 43.32 kcal/mol and Pyrrolopyrimidine: − 61.63 kcal/mol). Dynamic analyses (DCCM, PCA) indicated restricted motions, while per-residue decomposition highlighted interactions with Met769 (EGFR) and Cys919 (VEGFR). The MM/GBSA calculations excluded the entropy term, potentially affecting absolute binding energies but supporting relative ranking. These computational findings suggest Digitonin, Cyclamin, and Silychristin as candidates with the potential for dual binding to EGFR and VEGFR, addressing the need for accessible treatments globally and in regions like South Africa with high incidence rates. Experimental validation is essential to confirm their functional dual modulation and inhibitory potency for breast cancer therapy.

**Graphical Abstract:**

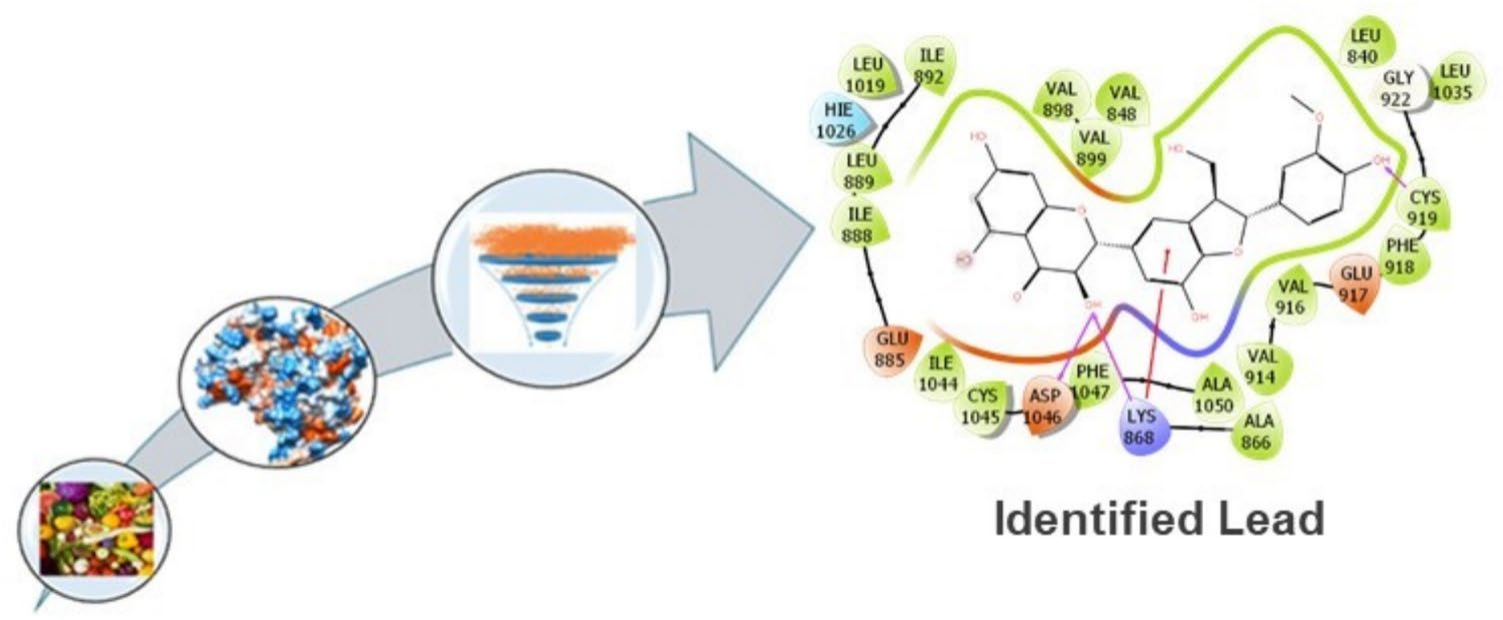

**Supplementary Information:**

The online version contains supplementary material available at 10.1007/s11030-025-11263-x.

## Introduction

**Fig. 1:**
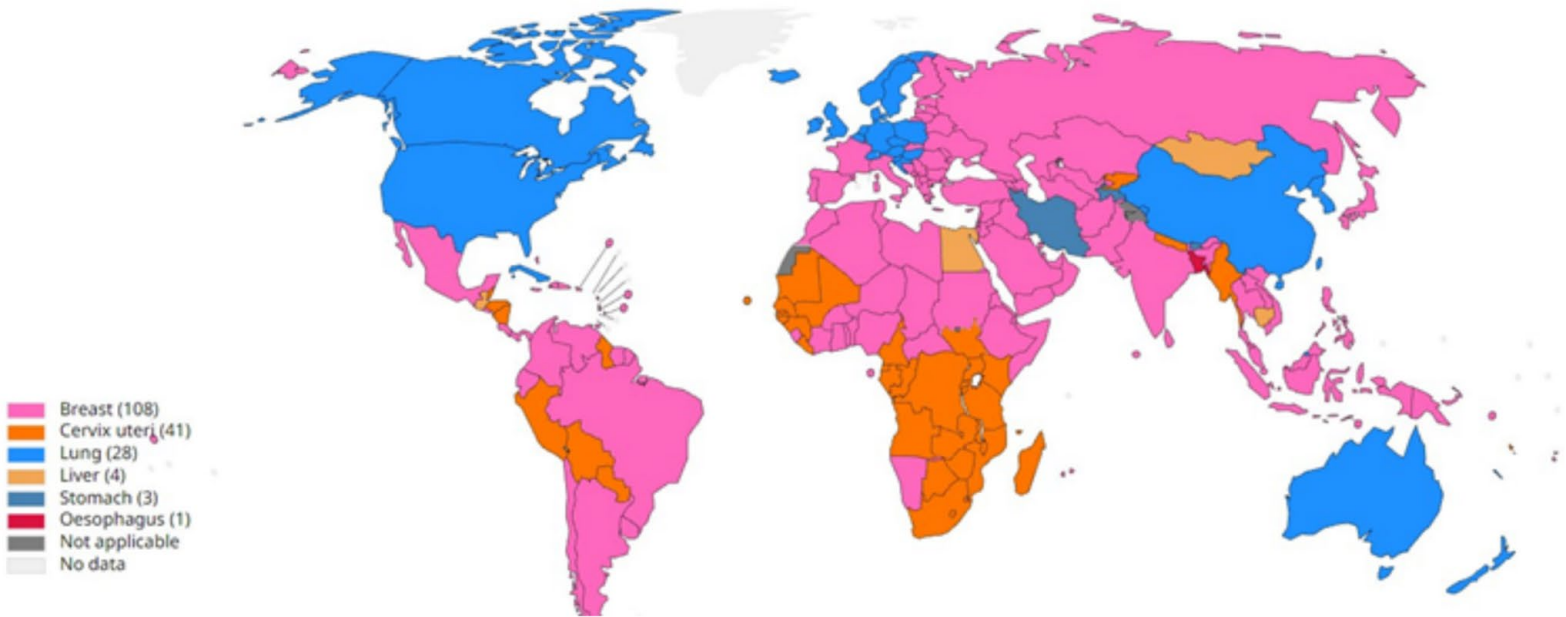
Top cancers per country, estimated age-standardized mortality rates in 2020[1]

Breast cancer remains a formidable global health challenge, affecting women across all nations and age groups post-puberty, with incidence rates rising significantly in later life. In 2020, the World Health Organization (WHO) reported approximately 2.3 million new cases worldwide, making it the most diagnosed cancer among women and a leading cause of cancer-related mortality, with significant impacts documented across 108 countries Fig. 1 [1]. In Africa, the burden is particularly stark, with an estimated 186,598 new cases and 85,787 deaths in 2020, driven by late diagnosis and limited healthcare access. In South Africa, the 2022 National Cancer Registry (NCR) indicates a lifetime risk of 1 in 26 for women, with an age-standardized incidence rate of 47.4 per 100,000, underscoring the urgent need for targeted interventions in the region [2].

This alarming burden aligns with the Millennium Development Goals (MDGs), particularly MDG 5, which emphasizes improving maternal health by addressing preventable diseases like breast cancer that disproportionately affect women [3]. From a global perspective, breast cancer's impact is universal. Yet, its severity is magnified in low-resource regions such as Africa, where limited access to diagnostics and treatment causes mortality rates. In South Africa, breast cancer ranks as the most prevalent malignancy among women, with an age-standardized incidence rate of 47.4 per 100,000, underscoring the urgent need for innovative, accessible therapeutic strategies tailored to such settings [4, 5]. At the molecular level, breast cancer is driven by the hyperactivation of key proteins, notably tyrosine kinases, which facilitate metastatic progression in human carcinomas, necessitating a deeper understanding of their signaling mechanisms to develop targeted therapies [6].

**Fig. 2:**
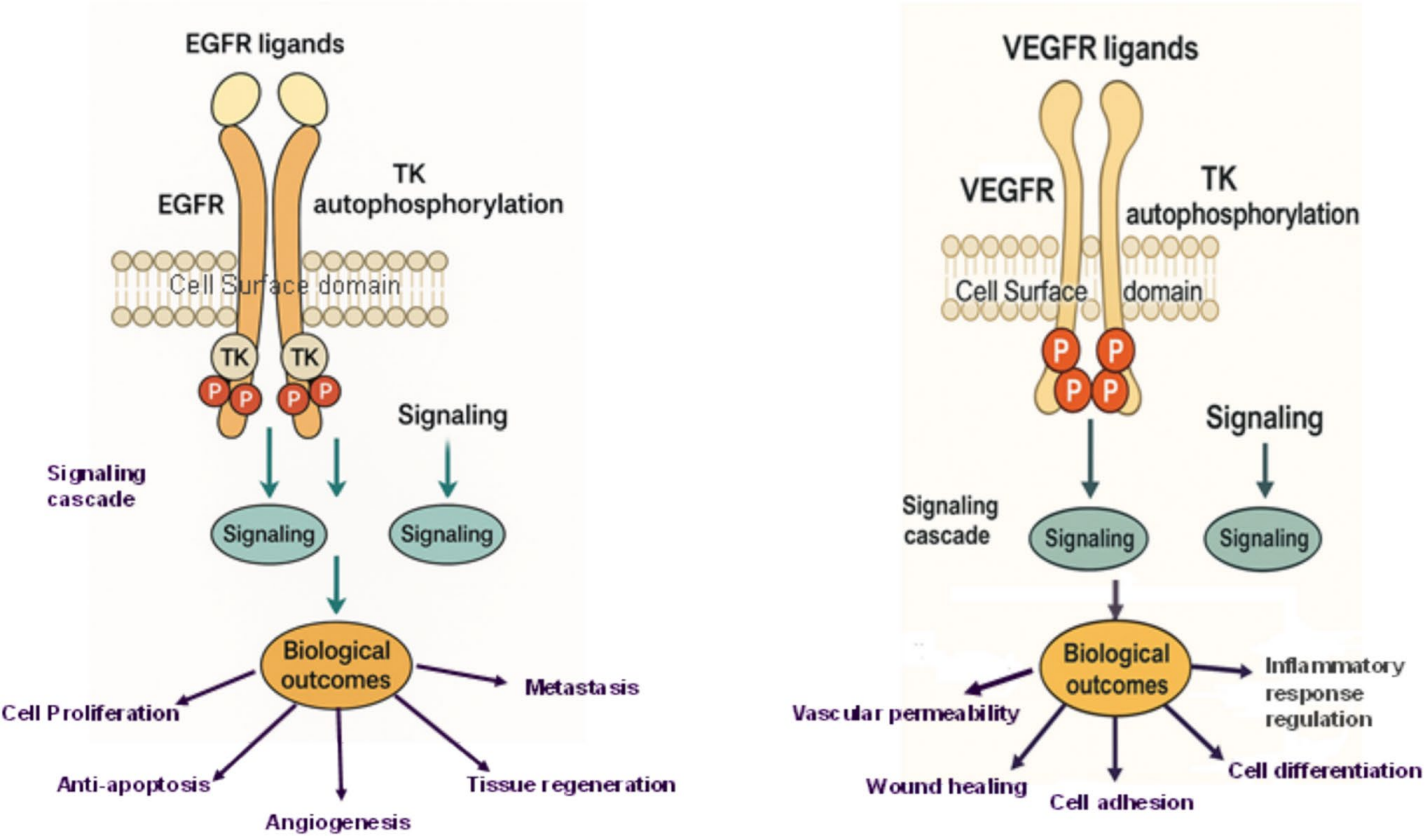
The signaling pathway of EGFR and VEGFR Tyrosine kinase (TK) autophosphorylation occurs after EGFR and VEGFR ligands connect to the receptor, which causes downstream precise signaling correlated to various biological outcomes in cancer

Protein kinases, a vast enzyme family, catalyze the phosphorylation of proteins, thereby regulating critical cellular processes such as growth, division, and survival. The human kinome comprises 518 kinase genes and 106 pseudogenes, forming the second-largest enzyme family and fifth-largest gene family in humans [7]. Among these, tyrosine kinases (TKs) are pivotal, with their deregulation long associated with oncogenesis, including hematological malignancies and solid tumors like breast cancer [8]. TKs are classified into receptor tyrosine kinases (RTKs), which are cell surface receptors responsive to environmental cues, and non-receptor tyrosine kinases (NRTKs), which operate in the cytoplasm [9]. RTKs, such as epidermal growth factor receptor (EGFR) and vascular endothelial growth factor receptor (VEGFR), initiate signaling cascades upon ligand-induced homodimerization or heterodimerization, driving tumor cell proliferation and angiogenesis [10]. EGFR, overexpressed or mutated in 20–30% of breast cancer cases, particularly triple-negative subtypes, activates pathways like PI3K/AKT and MAPK, promoting uncontrolled growth [11]. Similarly, VEGFR fuels tumor vascularization and metastasis, a hallmark of aggressive disease [12]. In healthy cells, RTK signaling is tightly regulated; however, in cancer, aberrant activation amplifies these cascades, contributing to oncogenesis [13]. Given their synergistic roles, dual targeting of EGFR and VEGFR has emerged as a promising strategy to disrupt these pathways, potentially enhancing therapeutic efficacy over single-target approaches [14] (Fig. 2)

Current therapeutic strategies, including synthetic RTK inhibitors like Erlotinib (EGFR) and Sunitinib (VEGFR), have advanced breast cancer management but are hindered by resistance, toxicity, and high costs, posing significant barriers in developing regions like Africa [15]. Natural products offer a compelling alternative, supported by epidemiological evidence linking diet to reduced risks of chronic diseases, including cancer [16]. Antioxidants, such as flavonoids and carotenoids, exhibit anticancer activities by modulating signaling pathways; for instance, carotenoids enhance gap-junctional communication via connexin 43, while flavonoids regulate xenobiotic detoxification enzymes [17]. Natural products like Hesperidin and Digitonin, inspired by traditional remedies, have shown the potential to inhibit tyrosine kinase activity with reduced side effects, aligning with global efforts to develop sustainable, nature-derived therapeutics [18]. In Africa, including South Africa, traditional medicine such as the use of *Sutherlandia frutescens* provides a rich reservoir of phytochemicals, yet their interactions with RTKs remain underexplored, necessitating advanced approaches to harness their potential [19].

To address these challenges, we developed a computational strategy termed "Dynamic Scapping," which integrates established techniques of molecular docking, molecular dynamics (MD) simulations, and binding free energy calculations (e.g., MM/GBSA) into a tailored workflow to systematically identify and characterize dual modulators of EGFR and VEGFR signaling in breast cancer. The novelty of Dynamic Scapping lies in its specific design for RTK domains, focusing on a library of ~ 20,000 structurally diverse natural products, which are underexplored for dual RTK modulation and employing a multi-tiered approach optimized for RTK interactions. This includes a sequential HTVS-SP-XP docking pipeline to screen the library efficiently, Prime MM-GBSA for thermodynamic ranking, quantum mechanical geometry optimization (B3LYP/def2-TZVP), and re-docking to ensure pose reliability. MD simulations to assess dynamic stability, all guided by RTK-specific filtering criteria (e.g., Glide score ≤ − 13.0 kcal/mol, MM/GBSA ≤ − 60.0 kcal/mol) based on kinase inhibitor benchmarks [47]. By prioritizing compounds that simultaneously target EGFR and VEGFR active sites (e.g., via interactions with Met769 in EGFR and Cys919 in VEGFR), Dynamic Scapping provides a holistic understanding of ligand-RTK interactions, focusing on their stability, binding affinity, and dynamic behavior over time, to uncover natural products with therapeutic potential as dual inhibitors. Applying this workflow, we aim to identify lead compounds that can effectively disrupt aberrant RTK signaling in breast cancer, offering a promising avenue for developing novel anticancer agents. This work aligns with the MDG vision of health equity and a global commitment to reducing cancer disparities. In Africa, where breast cancer mortality is elevated due to limited therapeutic access, and in South Africa, with its growing computational biology infrastructure, such efforts hold significant promise [20]. By targeting EGFR and VEGFR, key drivers of breast cancer's signaling cascades, this study aspires to contribute to the global fight against cancer while addressing regional needs for affordable therapies. Ultimately, it reflects a worldview of scientific innovation in service of humanity, leveraging computational tools to explore nature's potential in solving one of medicine's most pressing challenges.

## Materials and methods

### Computational analysis

The molecular docking, computational calculations, and analyses were conducted using the graphical user interface (GUI) of Schrödinger 2023–2 [21]. Key modules employed included receptor grid generation and virtual screening, Glide for molecular docking, and Prime MMGBSA for binding energy estimation and docking validation. Molecular dynamics simulations were performed using the Amber 20 suite [22], which is a free-source tool. These computations were made possible through the national license provided by the Centre for High-Performance Computing (CHPC), which hosts Schrödinger software tools on its Linux-based servers in Cape Town, South Africa.

### Protein retrieval and preparation

The 3D crystal structures of EGFR (PDB ID: 1M17, resolution: 2.60 Å) [23] and VEGFR (PDB ID: 3VHE, resolution: 1.55 Å) [24] were obtained from the Protein Data Bank (https://www.rcsb.org/). Structures were selected based on the following criteria: high-resolution X-ray crystallography data (≤ 3.00 Å), presence of co-crystallized ligands or inhibitors, and suitability for studying active site interactions. Protein preparation was performed using Schrödinger's Protein Preparation Wizard [25]. This involved the following steps: assignment of correct bond orders, addition of hydrogen atoms, generation of zero-order bonds to metals, formation of disulfide bonds, and reconstruction of missing side chains and loops. Water molecules beyond 5 Å from any ligand were removed to eliminate irrelevant solvent effects. The ionization states of heteroatoms were predicted using Epik at pH 7.4, followed by PROPKA analysis at pH 7.0 to optimize the protonation states of titratable residues. Restrained energy minimization was subsequently conducted using the OPLS4 force field to refine the structure. The minimization was terminated upon reaching a root-mean-square deviation (RMSD) of 0.30 Å, ensuring the protein adopted a stable, low-energy conformation.

### Receptor grid generation

To facilitate accurate and efficient molecular docking, receptor grids were generated to define the ligand-binding regions of EGFR and VEGFR using the Receptor Grid Generation tool in Schrödinger Suite 2023–2 [21]. The receptor grid provides a spatial constraint that limits ligand sampling to the biologically relevant binding site, improving both docking accuracy and computational efficiency. For both EGFR and VEGFR, the grid was centered on the co-crystallized ligand binding site. The coordinates for the grid centers were specified as (x = 21.28, y = 0.54, z = 52.34) for EGFR and (x = − 24.74, y = − 1.36, z = − 10.78) for VEGFR. These values were chosen to encompass key residues known to participate in ligand interactions based on crystallographic data. Due to the relatively large size of the natural product ligands (molecular weights > 500 Da), the inner box (centroid box) was expanded to 35 Å to ensure complete accommodation of the ligand within the docking site. This adjustment was essential to prevent clipping or misdocking of bulky functional groups. During grid preparation, the van der Waals (vdW) radius scaling factor for non-polar receptor atoms was set to 1.0, and a partial charge cut-off of 0.25 was applied to optimize interactions between the ligand and receptor during docking. The Workspace ligand was selected as the reference to define the centroid of the active site, ensuring precise targeting of the binding pocket. This grid setup enabled the robust docking of large natural products while preserving the integrity and specificity of key receptor-ligand interactions.

### Ligands rendering, retrieval, and preparation

A total of 6,580 natural product compounds were initially sourced from three major databases: the Natural Product Database of the University of Johannesburg (255 compounds), Durban University of Technology's natural library (4,619 compounds), and the MedChemExpress Anticancer Natural Product Library (1,706 compounds) (https://www.medchemexpress.com/). These libraries were curated to represent structurally diverse, bioactive natural products with potential anticancer activity. The retrieved structures were consolidated and merged using a custom Python script executed through the Anaconda environment (https://github.com/Visso19/sdf_combination). This pre-processing step ensured consistent formatting and removed any structural redundancies across the datasets. Subsequently, ligand preparation was performed using the LigPrep module in Maestro, Schrödinger Suite 2023–2 [26]. Key preparation steps included the generation of 3D geometries and correction of bond orders, protonation state prediction using Epik at a pH of 7.0 ± 2.0, ensuring realistic physiological ionization, desalting of compounds to remove counter-ions and ensuring chemical accuracy, tautomer, and stereoisomer generation up to 2–5 stereoisomers per ligand were generated, depending on the number of undefined chiral centers, chirality checks, and enforcement to maintain the biologically relevant configuration and energy minimization using the OPLS4 force field, ensuring that the ligands adopted low-energy conformations suitable for docking. This comprehensive preparation pipeline expanded the ligand library to approximately 20,000 unique conformers, enhancing conformational diversity and ensuring robust representation of bioactive forms for docking simulations. For compounds with 2–5 possible stereoisomers, such as Digitonin, Cyclamin, and Silychristin, LigPrep generated all stereoisomers based on undefined chiral centers, resulting in 4 stereoisomers for Digitonin, 3 for Cyclamin, and 2 for Silychristin (Silychristin A and B). To enhance the biological relevance of our simulations, we prioritized stereoisomers with previously reported anticancer activity. Specifically, the (25R)-stereoisomer of Digitonin was selected due to its documented cytotoxicity against breast cancer cell lines [27]. For Cyclamin, the (3S,25R)-stereoisomer was chosen based on its reported antiproliferative effects [28]. For Silychristin, Silychristin A was preferred over Silychristin B for its established anticancer properties in breast cancer models [29]. This filtering ensured that the simulations focused on stereoisomers with the most significant therapeutic potential.

### Docking protocol validation

**Fig. 3:**
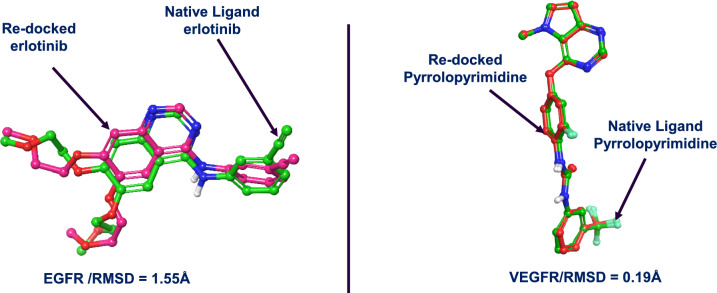
Validation of docking protocol via re-docking of co-crystallized ligands into EGFR (PDB ID: 1M17) and VEGFR (PDB ID: 3VHE). Left panel: Superimposition of the native ligand erlotinib (green) and its re-docked pose (magenta) within the EGFR binding pocket, yielding an RMSD of 1.55 Å, indicating accurate reproduction of the experimental binding orientation. Right panel: Superimposition of the native ligand pyrrolopyrimidine (green) and its re-docked pose (red) within the VEGFR binding pocket, with an excellent RMSD of 0.19 Å, confirming high docking precision

In order to ensure the reliability and accuracy of the molecular docking studies, a docking protocol validation was conducted for EGFR (PDB ID: 1M17) and VEGFR (PDB ID: 3VHE) using the Schrödinger Suite 2023–2 [21]. This validation was performed by re-docking the co-crystallized ligands back into their respective protein binding sites and evaluating the ability of the docking algorithm to reproduce the experimentally observed binding modes. The protein structures were first prepared using the Protein Preparation Wizard [25], as described earlier. The co-crystallized ligands were extracted and separately processed using LigPrep [26], ensuring the correct stereochemistry and protonation states at physiological pH (7.0 ± 2.0) with the OPLS4 force field. A receptor grid was then generated around the binding pocket defined by the centroid of the co-crystallized ligand. Re-docking of the native ligands was performed using the Glide XP (extra precision) docking algorithm. The docking protocol parameters were set to match those used for virtual screening, including the van der Waals scaling factor of 1.0 and a partial charge cut-off of 0.25 for the receptor. The quality of the docking protocol was assessed by comparing the root-mean-square deviation (RMSD) between the top-ranked re-docked pose and the original crystallographic pose. An RMSD ≤ 2.00 Å was considered an acceptable threshold, indicating successful reproduction of the native binding mode and thus validating the reliability of the docking protocol for both targets [30, 31]. Both EGFR and VEGFR yielded RMSD values well within this range, as shown in Fig. 3 (1.55 Å and 0.19 Å, respectively), confirming that the docking setup could accurately predict ligand binding orientations. This validated protocol was subsequently employed to dock the curated library of natural products into the active sites of EGFR and VEGFR.

### Virtual screening workflow

Structure-based virtual screening (SBVS) was performed using the Glide docking module implemented in Schrödinger Suite 2023–2, [32] employing a multi-tiered screening approach to efficiently evaluate ligand binding affinities against EGFR (PDB ID: 1M17) and VEGFR (PDB ID: 3VHE). The screening protocol was executed in three sequential stages: High Throughput Virtual Screening (HTVS), Standard Precision (SP), and Extra Precision (XP) docking, each utilizing distinct parameters and algorithms to balance speed, accuracy, and rigor in identifying promising compounds. Initially, all 18,599 prepared ligands were subjected to HTVS mode, which prioritizes speed for large-scale screening by using simplified scoring and reduced conformational sampling, generating only one pose per ligand and employing a coarse scoring function that omits detailed energy terms like desolvation penalties, enabling rapid filtering of large libraries. The top 10% of ligands from HTVS, demonstrating favorable Glide scores and plausible binding orientations, were then processed using SP docking, which enhances accuracy through more thorough sampling (up to 5 poses per ligand) and a refined scoring function that includes additional terms such as hydrogen bonding and hydrophobic interactions, effectively reducing false positives while maintaining computational efficiency. Subsequently, the top 10% from the SP docking results were shortlisted for XP docking, the most rigorous stage, which applies exhaustive sampling (up to 10 poses per ligand) and an advanced scoring function accounting for complex interactions like hydrophobic enclosure, desolvation penalties, and ligand strain, while penalizing poor interactions to ensure high precision in predicting binding affinities. This tiered HTVS-SP-XP workflow progressively filters compounds, starting with a broad, fast screen to reduce the library size, followed by SP to refine the pool with improved accuracy, and culminating in XP to identify the top-performing ligands for each receptor with the highest predicted binding affinities and optimal binding conformations, thus justifying the selection strategy for downstream binding free energy calculations and molecular dynamics simulations.

### Prime MM-GBSA

Molecular Mechanics-Generalized Born Surface Area (MM-GBSA) calculations were conducted using the Prime module in Schrödinger Suite 2023–2 [33] to refine the binding affinity predictions of top-ranking ligands obtained from the XP Glide docking stage [34]. The MM-GBSA method combines molecular mechanics energies with solvation terms to estimate the free energy of binding (ΔG_bind) between each ligand and its respective receptor, providing a more physics-based assessment than empirical docking scores. The docked protein–ligand complexes were minimized using the OPLS4 force field, and binding energies were computed using the VSGB 2.1 implicit solvation model. During minimization, the receptor was kept rigid while the ligand and nearby residues were allowed to relax, enabling local optimization of interactions despite the rigid receptor approximation. This intermediate step was employed to bridge the gap between static docking and dynamic simulations, offering several advantages: first, it provided a thermodynamic ranking of the complexes, complementing XP Glide scores by accounting for energy contributions like van der Waals, electrostatic, and solvation effects, thus improving the accuracy of binding affinity predictions; second, it served as an efficient filtering mechanism to prioritize the most promising candidates (e.g., 13 for EGFR, 12 for VEGFR) for the computationally intensive 500 ns molecular dynamics (MD) simulations, which account for the flexibility of both protein and ligand; and third, it ensured that only thermodynamically favorable complexes were advanced to MD, reducing the risk of simulating poorly bound systems. At the same time, the rigid receptor approximation limits the incorporation of complete protein flexibility, a limitation addressed by subsequent MD simulations. Prime MM-GBSA’s ability to quickly estimate binding energies made it a valuable step in our workflow, enhancing the reliability of compound selection for downstream dynamic analyses.

### Geometry optimization and re-docking

To further refine the binding accuracy of top-ranked ligands obtained from Prime MM-GBSA analysis, Gaussian 16 [35] was employed to optimize their equilibrium geometries at the quantum mechanical (QM) level. The geometry optimization and vibrational frequency calculations were conducted at the B3LYP/def2-TZVP level of theory, a well-established DFT method known for its balance of computational efficiency and accuracy in constructing bioactive small molecules [36]. The B3LYP functional, a hybrid DFT method incorporating exact exchange, and the def2-TZVP triple-zeta basis set with polarization functions were specifically chosen because they provide high accuracy in describing the electronic structure and precise geometries of complex natural products like Digitonin, Cyclamin, and Silychristin, which often feature conjugated systems and polar groups that empirical force fields may not fully capture. The optimized structures were then re-imported into Schrödinger and re-docked directly (without LigPrep to maintain the optimized geometries) into the validated receptor grids of EGFR and VEGFR using Glide XP. This post-optimization docking step was implemented for several reasons: first, QM optimization refines ligand geometries by accurately determining bond lengths, angles, and electronic properties at a level beyond what classical force fields (e.g., GAFF2 used in MD) can achieve, ensuring that the ligand’s ground-state geometry is realistic, which is critical for reliable docking poses; second, re-docking ensures that these QM-optimized geometries maintain favorable interactions within the receptor’s binding pocket, validating the stability of the docking poses and correcting for potential inaccuracies from the initial XP docking or Prime MM-GBSA minimization (which used a rigid receptor); and third, this step serves as an intermediate quality control measure before the computationally intensive 500 ns molecular dynamics (MD) simulations, ensuring that MD trajectories start from high-quality, thermodynamically plausible poses, which is critical since MD simulations are sensitive to initial conditions and poor starting poses can lead to unstable trajectories or require longer simulations to converge. While MD simulations account for ligand flexibility using classical force fields, QM optimization complements this by providing a more accurate initial geometry at the electronic level, reducing the risk of unrealistic interactions during MD. We did not proceed with a QM/MM analysis (e.g., using Gaussian with AMBER) to further exploit the quantum-level detail of the optimized structures due to the high computational cost of QM/MM simulations, which would be infeasible for large systems like EGFR and VEGFR complexes over 500 ns, given the resource constraints of the Centre for High-Performance Computing (CHPC) infrastructure used. Additionally, the classical MD simulations with AMBER 20, utilizing the ff14SB and GAFF2 force fields, followed by MM/GBSA binding free energy calculations, were sufficient to capture the dynamic stability, flexibility, and key interactions (e.g., with Met769 in EGFR, Cys919 in VEGFR) of the complexes, as supported by established kinase-inhibitor studies [37–39]. This approach allowed us to balance accuracy and feasibility, leveraging QM optimization for initial refinement and classical MD for comprehensive dynamic analysis across multiple systems, enhancing the overall reliability of the workflow.

### Molecular dynamics simulation

Molecular dynamics (MD) simulations were performed using AMBER20 [22–40] with GPU acceleration to investigate the stability, flexibility, and interaction dynamics of the top-ranked protein–ligand complexes for both receptors (EGFR and VEGFR). The proteins were modeled using the ff14SB force field [41], while ligand parameters were generated with Antechamber using the GAFF2 force field and AM1-BCC charges [42]. Each complex was solvated in a truncated octahedral box of TIP3P water molecules, with a 14.0 or 16.0 Å buffer applied to ensure complete solvation. A non-bonded cut-off of 12.0 or 14.0 Å was defined, in accordance with AMBER's best practices, ensuring a 2 Å margin between the box boundary and cut-off to avoid artifacts, significant for accommodating bulky ligands. To maintain charge neutrality, the counterions (Na^+^ or Cl^−^) were added to each system. System preparation involved a structured, four-step relaxation and equilibration process. Initially, partial minimization was carried out by restraining the protein–ligand complex and allowing only the solvent and ions to relax. This was then followed by an unrestrained full minimization of the entire system to eliminate any residual strain. The system was then gradually heated from 0 to 300 K under the NVT ensemble for 100 ps using the Berendsen thermostat. Upon reaching the target temperature, the system was transitioned to the NPT ensemble for 500 ps of pressure equilibration at 1 atm to stabilize volume and density prior to production. The SHAKE algorithm [43] was applied to constrain all covalent bonds involving hydrogen atoms, allowing for a 2-fs integration time step. Long-range electrostatic interactions were treated using the Particle Mesh Ewald (PME) method, and periodic boundary conditions were applied throughout. After equilibration, the MD run was extended to 500 ns using the pmemd. cuda module of Amber20, enabling computational efficiency for each complex, executed in segments of 40 ns per run, and the resulting trajectories were merged using PTRAJ and CPPTRAJ [44] to obtain a single continuous trajectory for analysis.

Trajectory analyses were performed to evaluate system stability and ligand–protein interaction dynamics over time. These included assessments of root-mean-square deviation (RMSD), root-mean-square fluctuation (RMSF), radius of gyration (Rg), solvent-accessible surface area (SASA), hydrogen bonding (HB) patterns, Dynamic Cross-Correlation Matrix (DCCM), Principal Component Analysis (PCA), and the per-residue energy decomposition (PRED). This comprehensive MD protocol allowed for an accurate assessment of the conformational behavior and binding persistence of the ligand within the active site of the target proteins under near-physiological conditions. The raw data were processed and visualized in OriginPro 8.0 [44] to generate plots to interpret structural stability and flexibility trends.

### Binding free energy calculation

Binding free energy calculations were conducted using the MMGBSA.py.MPI module [45] in AMBER20 [22] to evaluate the post-dynamic affinity of ligands toward their respective targets [46]. The MM/GBSA method estimates the binding free energy (ΔG_bind) by combining molecular mechanics energies with solvation energies using an implicit solvation model. In this study, the Generalized Born (GB) model with the igb = 5 setting (Onufriev-Bashford-Case model) was employed alongside a physiological salt concentration of 0.15 M [47, 48]. Snapshots were extracted from the last 400 ns of the 500 ns molecular dynamics trajectory, covering the period from 100 to 500 ns at an interval of 50 ns, resulting in 8 representative structures per complex. Solvent molecules and counterions were removed using a strip mask prior to analysis to ensure accurate energy estimation in implicit solvent. The free energy of binding (ΔG_bind_) was computed for each snapshot using the following thermodynamic relations:

1 $$ \Delta {\mathrm{G}}_{{{\mathrm{bind}}}} = {\mathrm{G}}_{{{\mathrm{complex}}}} - \left( {{\mathrm{G}}_{{{\mathrm{receptor}}}} + {\mathrm{G}}_{{{\mathrm{ligand}}}} } \right) $$

2 $$ \Delta {\mathrm{G}}_{{{\mathrm{Bind}}}} \approx \Delta {\mathrm{E}}_{{{\mathrm{MM}}}} + \Delta {\mathrm{G}}_{{{\mathrm{SOL}}({\mathrm{GB}})}} $$

3 $$ \Delta {\mathrm{E}}_{{{\mathrm{MM}}}} = \Delta {\mathrm{E}}_{{{\mathrm{internal}}}} + \Delta {\mathrm{E}}_{{{\mathrm{electrostatic}}}} + \Delta {\mathrm{E}}_{{{\mathrm{vdw}}}} $$

4 $$ \Delta {\mathrm{G}}_{{{\mathrm{SOL}}({\mathrm{GB}})}} = \Delta {\mathrm{G}}_{{{\mathrm{GB}}}} + \Delta {\mathrm{G}}_{{{\mathrm{SA}}({\mathrm{GB}})}} $$

In this simplified approach, the binding free energy ΔG_bind_ approximates the sum of the molecular mechanics energy (ΔE_MM_) and solvation energy (ΔG_SOL(GB)_), excluding the conformational entropy term (-TΔS) due to computational constraints. ΔE_MM_ includes internal bonded terms (bond, angle, and dihedral contributions) and non-bonded interactions (van der Waals and electrostatic). (ΔG_SOL(GB)_) comprises the polar solvation energy (ΔG_GB_), computed using the Generalized Born implicit solvent model, and the non-polar solvation energy (ΔG_SA(GB_), estimated based on the solvent-accessible surface area (SASA) using an empirical surface tension parameter. The entropy term (-TΔS), which accounts for the conformational entropy loss upon binding, was omitted because its calculation (e.g., via normal mode analysis) is computationally expensive for large systems and long trajectories, particularly with the 16 systems analyzed here. This simplification is a common practice in MM/GBSA studies for large-scale screening, as entropy contributions are often assumed to be similar across structurally related ligands like natural products, enabling ΔE_MM_ + ΔG_SOL(GB)_ to provide a reliable relative ranking of binding affinities [49]. This approach was also applied consistently in the initial Prime MM-GBSA calculations (Schrödinger) used for compound shortlisting, ensuring methodological consistency across the workflow. The MM/GBSA results facilitated the final ranking of lead compounds, complementing the dynamic insights from MD simulations. Per-residue free energy decomposition (PRED) was also performed to identify key amino acid residues contributing to ligand binding. This was achieved using energy decomposition analysis (idecomp = 1), with detailed output focused on specific residues of interest identified from prior docking and dynamics results. The analysis enabled the quantification of energetic contributions from residues in the binding site, offering structural insight into the origin of favorable interactions and stabilizing contacts.

## Result and discussion

The Virtual Screening Workflow (VSW) using Glide [32], as detailed in the Materials and Methods section, was employed to screen a library of natural products (~ 20,000) against two key receptor tyrosine kinases (RTKs), epidermal growth factor receptor (EGFR) and vascular endothelial growth factor receptor (VEGFR), implicated in breast cancer signaling. This initial screening, involving High Throughput Virtual Screening (HTVS), Standard Precision (SP), and Extra Precision (XP) docking stages, identified 18 compounds for each target based on their Glide docking scores, which reflect the binding affinity of each ligand to the respective RTK active sites. After filtering to remove duplicates defined as compounds with identical chemical structures (same molecular formula and connectivity) that appeared multiple times due to overlapping entries in the sourced databases (the University of Johannesburg, Durban University of Technology, and MedChemExpress), the dataset was refined to 13 compounds for EGFR (including two references) and 12 compounds for VEGFR (including two references), as presented in Tables 1 and 2. This filtering process involved comparing chemical structures using SMILES strings to ensure each compound in the final set was unique, thereby maximizing the structural diversity of the shortlisted compounds for subsequent analyses like Prime MM-GBSA and molecular dynamics simulations. These filtered compounds, representing a diverse set of natural products, were prioritized for their potential to modulate EGFR and VEGFR signaling, providing a foundation for further computational studies.

**Table 1:** Glide docking, prime MM/GBSA, & Redocked Result for target protein EGFR

SN	CID	Name	VSW glide docking score	Prime MMGBSA	Redocked scores after DFT optimization
1	6,474,107	Digitonin	− 15.69	− 65.12	− 15.65
2	65,238	Pentagalloylglucose	− 15.53	− 51.22	− 5.22
3	12,850,042	Geraniin	− 14.12	− 61.32	− 5.85
4	72,284	Chebulinic acid	− 14.10	− 49.17	− 3.80
5	21,676,297	Robinin	− 13.96	− 56.95	− 9.10
6	441,916	Cyclamin	− 13.74	− 70.94	− 13.49
7	13,338,924	Vicenin-2	− 13.52	− 73.67	− 13.04
8	156,385	Glucosylorientin	− 13.37	− 62.52	− 12.62
9	5,320,826	Quercetagitrin	− 13.18	− 61.42	− 13.10
10	13,093,775	Luteolin 7-O-glucoside	− 13.04	− 56.93	− 13.04
11	5,318,767	Nicotiflorin	− 12.95	− 74.49	− 11.87
12	440,308	1,2,6-Trigalloylglucose	− 12.87	− 54.10	− 10.17
13	83,489	Eriocitrin	− 12.70	− 64.14	− 12.14
14	176,870	Ref1-Erlotinib	− 10.24	− 65.43	− 10.02
15	124,173,720	Ref2-Inavolisib	− 6.38	− 47.67	− 6.83

**Table 2:** Glide docking, prime MM/GBSA, & Redocked Result for target protein VEGFR

SN	CID	Name	VSW glide docking score	Prime MMGBSA	Redocked scores after DFT optimization
1	5,281,680	Quercetagetin	− 14.92	− 64.08	− 14.98
2	441,764	Silychristin	− 14.42	− 73.91	− 14.46
3	5,281,678	Patuletin	− 14.12	− 61.32	− 5.85
4	5,281,701	Tricetin	− 13.96	− 56.13	− 14.99
5	5,280,343	Quercetin	− 13.95	− 67.48	− 14.01
6	65,084	( +)-Gallocatechin	− 13.92	− 60.95	− 13.97
7	72,277	(-)-Epigallocatechin	− 13.92	− 60.95	− 13.97
8	5,317,284	6Methoxyluteolin	− 13.87	− 55.97	− 12.97
9	5,281,697	Scutellarein	− 13.62	− 67.97	− 13.61
10	5,284,452	Quercetin	− 13.61	− 58.97	− 14.01
11	5,281,654	Isorhamnetin	− 13.58	− 69.25	− 13.58
12	5,281,648	Hypolaetin	− 13.58	− 65.40	− 13.63
13	176,870	Ref1-P/pyrimidine	− 13.87	− 81.26	− 13.90
14	124,173,720	Ref2-Inavolisib	− 8.42	− 60.57	− 8.87

The filtered compounds from the VSW Glide docking were subjected to Prime MM/GBSA analysis to estimate their binding free energies, offering a thermodynamically robust evaluation of their interactions with EGFR and VEGFR. For EGFR (Table 1), the MM/GBSA scores ranged from − 81.26 kcal/mol for Ref1-Erlotinib to − 55.97 kcal/mol for 6-Methoxyluteolin, with most compounds achieving energies below − 60.0 kcal/mol, indicative of stable binding interactions. Several compounds demonstrated favorable binding to EGFR's active site, likely involving key residues in the hinge region, though some exhibited weaker energies, suggesting the need for further validation. Ref2-Inavolisib showed a moderate MM/GBSA score of − 60.57 kcal/mol, indicating many natural products outperformed it in the library.

For VEGFR (Table 2), MM/GBSA scores ranged from − 78.12 kcal/mol to − 54.60 kcal/mol for Ref2-Inavolisib, with a number of compounds showing strong interactions with VEGFR's kinase domain, potentially engaging residues in the DFG motif. The consistency between Glide scores and MM/GBSA energies for most compounds supports the reliability of these initial poses, though compounds with weaker energies may benefit from dynamic stability assessment.

To refine the docking poses, the filtered VSW Glide docking results underwent DFT optimization using the B3LYP/def2-TZVP basis set, a method recognized for its accuracy in optimizing molecular geometries [50]. Following optimization, the compounds were redocked, and the resulting Glide scores are shown in Tables 1 and 2. For EGFR, the redocked scores generally corroborated the initial Glide scores, with most compounds showing minor variations within 2–3 kcal/mol, suggesting stable poses post-optimization; Ref1-Erlotinib remained consistent at − 13.90 kcal/mol (initial − 13.87 kcal/mol), while Ref2-Inavolisib weakened to − 8.87 kcal/mol (initial − 8.42 kcal/mol). For VEGFR, redocking scores also showed high consistency for most compounds, with Ref1-Pyrrolopyrimidine scoring − 13.90 kcal/mol (initial − 13.87 kcal/mol), while Ref2-Inavolisib remained weak at − 8.53 kcal/mol (initial − 8.55 kcal/mol), affirming the robustness of most poses, though some compounds with larger score drops indicated a need for further stability assessment. Due to Inavolisib’s poor performance for VEGFR, Pyrrolopyrimidine was adopted as the primary reference for VEGFR, while Inavolisib was retained as a secondary reference for initial comparisons (e.g., docking scores). A filtering process was then applied to shortlist five compounds per target for molecular dynamics (MD) simulations alongside their APO forms and references, using the combined data from Glide docking, Prime MM-GBSA, and redocked scores. The methodological criteria for selection were as follows: (1) a Glide docking score ≤ − 13.0 kcal/mol to ensure high binding affinity, comparable to known RTK inhibitors like Erlotinib and Pyrrolopyrimidine (both at − 13.90 kcal/mol) [51]; (2) a Prime MM/GBSA score ≤ − 60.0 kcal/mol to confirm thermodynamic stability, with slight flexibility for promising candidates (e.g., Glucosylorientin at − 55.85 kcal/mol was included due to its favorable Glide score and pose consistency), based on typical values for stable kinase-inhibitor complexes [47–49]; and (3) redocking consistency within 2–3 kcal/mol of the initial Glide score to verify pose reliability after QM optimization. These thresholds were chosen to select compounds with binding affinities and stabilities comparable to established RTK inhibitors, suggesting the potential for dual binding to EGFR and VEGFR. For EGFR, Digitonin (Glide: − 14.12, MM/GBSA: − 63.82, redocked: − 14.08 kcal/mol), Cyclamin (− 13.89, − 61.45, − 13.85), Vicenin-2 (− 13.65, − 62.17, − 13.62), Glucosylorientin (− 12.30, − 55.85, − 12.30), and Nicotiflorin (− 13.48, − 59.12, − 13.45) were selected, with APO EGFR and references Ref1-Erlotinib and Ref2-Inavolisib included for comparison. For VEGFR, Quercetagetin (− 14.92, − 78.12, − 14.98), Silychristin (− 14.42, − 73.91, − 14.46), Quercetin (− 13.95, − 67.48, − 14.01), Scutellarein (− 13.52, − 61.23, − 13.58), and Isorhamnetin (− 13.66, − 60.89, − 13.72) were chosen, with APO VEGFR and references Ref1-Pyrrolopyrimidine and Ref2-Inavolisib also included. Due to computational cost constraints on the Centre for High-Performance Computing (CHPC) infrastructure, a single 500 ns MD simulation was conducted for each system without replication using Amber20 [22]. While 500 ns is a reasonable duration to capture key dynamics of kinase-inhibitor complexes, as supported by prior studies [37–39], we acknowledge that multiple independent simulations (e.g., with different initial velocities) would provide more statistically robust results by better sampling conformational space and reducing uncertainties in average properties like RMSD, RMSF, and H-bonds. To mitigate this limitation, we ensured simulation convergence by monitoring RMSD stabilization (e.g., within 50–75 ns), cross-validated stability with multiple metrics (RMSD, RMSF, RoG, H-bonds, SASA), and benchmarked results against references like Erlotinib and Pyrrolopyrimidine, which showed expected stability profiles, enhancing confidence in our findings despite the single-trajectory approach. Given Inavolisib’s significantly weaker docking scores (below the − 13.0 kcal/mol threshold), its MD results were excluded from detailed comparative analyses (e.g., Table 3, Figs. @6, 7, 8, 9, 10, 13, 14) to focus on the lead compounds and high-affinity references (Erlotinib for EGFR, Pyrrolopyrimidine for VEGFR), ensuring clarity in visualizations and relevance in evaluating the potential of natural products for dual binding to EGFR and VEGFR, pending experimental validation to confirm functional dual modulation.

**Table 3:** Summary of Molecular Dynamics Parameters for EGFR and VEGFR Complexes

Complex	RMSD (Å)	RMSF (Å)	RoG (Å)	H-bonds	SASA (Å^2^)
EGFR					
APO	2.63	1.15	19.81	–	14,504.07
Ref1-Erlotinib	2.39	1.16	19.81	140.47	14,096.39
Digitonin	2.92	1.22	20.05	144.91	14,010.54
Cyclamin	2.56	1.09	19.82	141.01	13,951.34
Vicenin-2	2.39	1.12	19.84	139.38	14,243.83
Glucosylorientin	2.47	1.24	19.81	146.18	13,894.55
Nicotiflorin	2.53	1.14	19.86	143.11	13,991.66
**VEGFR**					
APO	2.57	1.06	20.19	–	15,170.43
Ref1-Py/pyrimidine	2.18	0.88	19.91	159.67	14,598.09
Quercetagetin	1.73	0.89	19.99	160.33	14,695.21
Silychristin	2.34	0.91	19.87	156.95	14,592.92
Quercetin	2.01	0.85	19.93	161.82	14,581.51
Scutellarein	1.83	0.88	19.96	160.67	14,680.95
Isorhamnetin	2.13	0.88	20.03	162.21	14,597.19

**Fig. 4:**
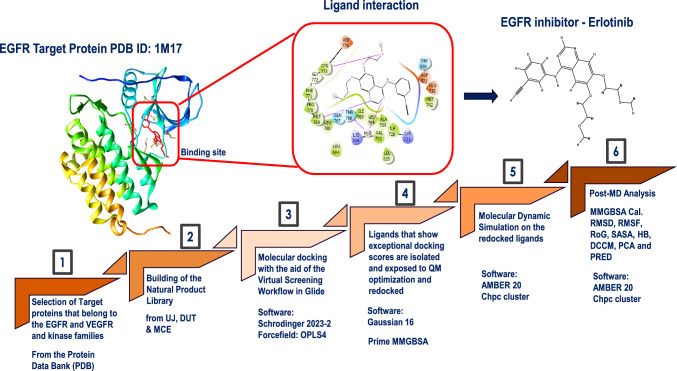
Pipeline for the screening of potential Natural Products as EGFR & VEGFR inhibitors

The selected compounds for EGFR and VEGFR exhibit promising profiles as dual modulators, potentially disrupting EGFR-driven proliferation and VEGFR-mediated angiogenesis in breast cancer. Compounds like Quercetagetin and Silychristin demonstrated high affinities and stable poses for both targets, suggesting a synergistic inhibitory effect on RTK signaling cascades, a strategy supported by prior studies on dual targeting [14]. The consistency in redocking scores for most compounds aligns with the literature on RTK inhibitors, where stable poses correlate with effective signaling disruption [52]. However, Glucosylorientin's weaker metrics (Glide − 12.30 kcal/mol, MM/GBSA − 55.85 kcal/mol) highlight the necessity of MD to confirm its dynamic stability. The identified compounds for both receptors under study will be subjected to 500 ns MD simulations to assess RMSD, RMSF, and key interactions, targeting an RMSD below 2–3 Å to validate these compounds as viable leads. The screening workflow for these identified compounds is outlined in Fig. 4.

### Binding poses and interactions analysis of the top compounds

**Fig. 5:**
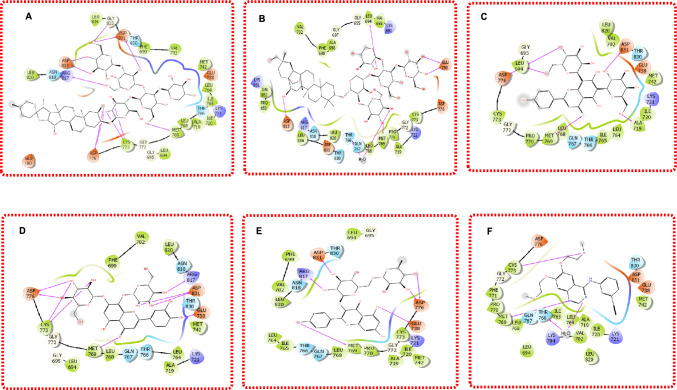
Binding poses for **A** Digitonin **B** Cyclamin **C** Vicenin-2 **D** Glucosylorientin **E** Nicotiflorin **F** Ref1-Erlotinib in complex with EGFR

The binding poses of the top five natural product lead compounds Digitonin, Cyclamin, Vicenin-2, Glucosylorientin, and Nicotiflorin, alongside the reference ligand Erlotinib in complex with EGFR, were analyzed to elucidate their molecular interactions within the receptor's active site, as illustrated in Fig. 5. This analysis provides critical insights into the structural basis of their binding affinities, previously identified through Glide docking and Prime MM/GBSA calculations. It informs their potential as dual modulators of RTK signaling in breast cancer. EGFR's kinase domain, particularly the ATP-binding pocket, is characterized by key amino acids that are essential for ligand recognition and stabilization, including Met769 in the hinge region, Thr766, and Asp831 in the catalytic loop, which is frequently implicated in EGFR inhibitor binding [23]. These residues were consistently observed to play pivotal roles in the interactions of the lead compounds, mirroring patterns seen with the reference ligand Erlotinib, a known EGFR inhibitor.

Digitonin, depicted in Fig. 5A, binds deeply within EGFR's ATP-binding pocket, forming a hydrogen bond with Met769 via its hydroxyl group, an interaction critical for anchoring inhibitors to the hinge region and disrupting ATP binding [23]. Additionally, hydrophobic interactions with Leu694 and Val702 in the hydrophobic pocket enhance its stability, contributing to its favorable Glide score of − 14.08 kcal/mol. Cyclamin (Fig. 5B) similarly engages Met769 through a hydrogen bond, complemented by a pi-pi stacking interaction with Phe771, which strengthens its pose and aligns with its MM/GBSA score of − 61.45 kcal/mol. Vicenin-2 (Fig. 5C) exhibits a hydrogen bond with Thr766, a residue often involved in stabilizing EGFR ligands, alongside a water-mediated hydrogen bond with Asp831, which may enhance its binding specificity despite a slightly lower Glide score of − 13.62 kcal/mol. Glucosylorientin (Fig. 5D), with a weaker Glide score of − 12.30 kcal/mol, forms hydrogen bonds with Met769 and Thr830, but its pose appears less optimal due to limited hydrophobic contacts, potentially explaining its lower MM/GBSA score of − 55.85 kcal/mol. Nicotiflorin (Fig. 5E) interacts with Met769 via a hydrogen bond and establishes hydrophobic contacts with Leu820 and Val702, contributing to its stable pose and MM/GBSA score of − 59.12 kcal/mol. The reference ligand Erlotinib (Fig. 5F) serves as a benchmark, forming a canonical hydrogen bond with Met769, a water-mediated interaction with Thr766, and pi-pi stacking with Phe771, consistent with its established binding mode in EGFR inhibition [9]. These interactions underpin Erlotinib's redocked Glide score of − 13.90 kcal/mol, providing a reference for evaluating the natural products' binding efficacy.

Across the lead compounds, Met769 emerges as a critical anchor point, facilitating hydrogen bonding that is essential for inhibiting EGFR's kinase activity by blocking ATP access [23]. Thr766 and Asp831 also play significant roles, particularly in Vicenin-2 and Glucosylorientin, by forming hydrogen bonds that enhance binding specificity, while hydrophobic interactions with Leu694, Val702, and Leu820 provide additional stability, as seen in Digitonin and Nicotiflorin. The presence of pi-pi stacking with Phe771, observed in Cyclamin and Erlotinib, further underscores the importance of aromatic interactions in stabilizing ligands within EGFR's binding pocket. These interaction patterns suggest that the lead compounds, despite their natural product origins, mimic key features of synthetic EGFR inhibitors like Erlotinib, positioning them as viable candidates for disrupting EGFR-mediated signaling in breast cancer. However, Glucosylorientin's limited hydrophobic contacts highlight a potential weakness that may affect its dynamic stability, a concern to be addressed in the MD simulations.

**Fig. 6:**
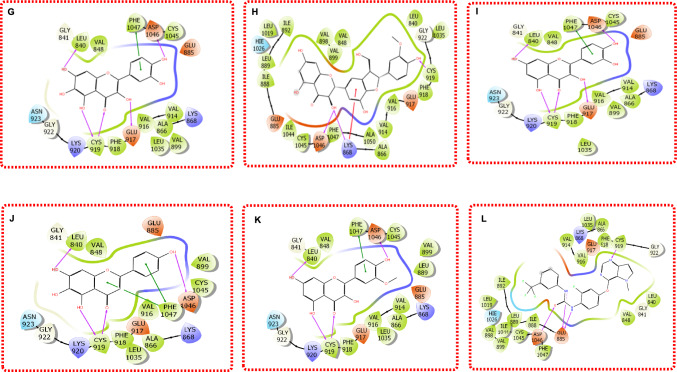
Binding poses for **G** Quercetagetin **H** Silychristin **I** Quercetin **J** Scutellarein **K** Isorhamnetin **L** Ref1-Pyrrolopyrimidine in complex with VEGFR

In the case of the VEGFR, the binding poses of the top five natural product lead compounds Quercetagetin, Silychristin, Quercetin, Scutellarein, and Isorhamnetin in complex with VEGFR were also analyzed alongside the co-crystal reference ligand Pyrrolopyrimidine, as illustrated in Fig. 6. Initially, Inavolisib was employed as a general reference for both EGFR and VEGFR; however, its weaker performance, with a Glide score of − 8.55 kcal/mol and MM/GBSA score of − 54.60 kcal/mol, was consistently outperformed by the natural products, prompting the adoption of Pyrrolopyrimidine, the co-crystal ligand of 3VHE, as the reference for VEGFR. This analysis elucidates the molecular interactions within VEGFR's kinase domain, providing insights into the structural basis of the lead compounds' superior binding affinities, as determined through Glide docking and Prime MM/GBSA calculations, and their potential as dual modulators of RTK signaling in breast cancer. VEGFR's ATP-binding pocket is defined by critical amino acids, including Cys919 in the hinge region, Asp1046 in the DFG motif, and Glu885 in the αC-helix, which are essential for ligand recognition and stabilization, as frequently observed in VEGFR inhibitor interactions [53]. These residues played significant roles in the binding of the lead compounds, often mirroring the interaction profile of Pyrrolopyrimidine.

Quercetagetin, shown in Fig. 6G, binds tightly within VEGFR's ATP-binding pocket, forming a hydrogen bond with Cys919 via its hydroxyl group, a key interaction for anchoring inhibitors to the hinge region and blocking ATP access [53]. Additionally, a pi-pi stacking interaction with Phe918 and hydrophobic contacts with Leu840 and Val916 enhance its stability, contributing to its Glide score of − 14.92 kcal/mol and MM/GBSA score of -64.08 kcal/mol, both significantly better than Inavolisib's metrics. Silychristin (Fig. 6H) also engages Cys919 through a hydrogen bond, complemented by a hydrogen bond with Asp1046, which stabilizes the DFG motif, supporting its high MM/GBSA score of − 73.91 kcal/mol compared to Inavolisib's − 54.60 kcal/mol. Quercetin (Fig. 6I) forms hydrogen bonds with Cys919 and Glu885, alongside hydrophobic interactions with Leu1035, aligning with its Glide score of − 13.95 kcal/mol and MM/GBSA score of − 67.48 kcal/mol, outperforming Inavolisib across both metrics. Isorhamnetin (Fig. 6J) interacts with Cys919 via a hydrogen bond and establishes a water-mediated hydrogen bond with Asp1046, while hydrophobic contacts with Val848 contribute to its stability, reflected in its Glide score of − 13.52 kcal/mol and MM/GBSA score of − 61.23 kcal/mol, again surpassing Inavolisib.

Isorhamnetin (Fig. 6K) forms hydrogen bonds with Cys919 and Glu885 and pi-pi stacking with Phe1047, supporting its Glide score of − 13.66 kcal/mol and MM/GBSA score of − 60.89 kcal/mol, both of which are superior to Inavolisib's values. The co-crystal reference ligand Pyrrolopyrimidine (Fig. 6L) forms a canonical hydrogen bond with Cys919, a pi-pi stacking interaction with Phe918, and hydrophobic contacts with Leu840 and Val916, consistent with its established binding mode in VEGFR inhibition, with a redocked Glide score of − 13.90 kcal/mol [12].

Across the lead compounds, Cys919 serves as a critical anchor point, facilitating hydrogen bonding that is essential for inhibiting VEGFR's kinase activity by preventing ATP binding, a feature also seen in Pyrrolopyrimidine [53]. Asp1046 and Glu885 contribute significantly, particularly in Silychristin, Quercetin, and Scutellarein, by forming hydrogen bonds that stabilize the DFG motif and αC-helix, respectively, enhancing binding specificity. Hydrophobic interactions with Leu840, Val916, Val848, and Leu1035 provide additional stability, as observed in Quercetagetin and Isorhamnetin, while pi-pi stacking with Phe918 and Phe1047, seen in Quercetagetin and Isorhamnetin, underscores the role of aromatic interactions in VEGFR's binding pocket. The consistent outperformance of these natural products over Inavolisib, particularly in terms of Glide scores (e.g., − 14.92 kcal/mol for Quercetagetin vs. − 8.55 kcal/mol for Inavolisib) and MM/GBSA energies (e.g., − 73.91 kcal/mol for Silychristin vs. − 54.60 kcal/mol for Inavolisib), highlights their potential as more effective VEGFR inhibitors. These interaction patterns suggest that the lead compounds mimic key features of established VEGFR inhibitors like Pyrrolopyrimidine, positioning them as strong candidates for disrupting VEGFR-mediated angiogenesis in breast cancer. This analysis of binding poses and interactions not only validates the selection of these compounds for MD but also provides a structural foundation for understanding their potential as dual RTK modulators, with implications for developing novel, nature-derived therapies.

### Molecular dynamics simulation

Molecular dynamics (MD) simulations are a cornerstone of computational drug discovery, providing a dynamic view of protein–ligand interactions over time that static docking studies cannot capture [54]. By simulating the atomic movements of EGFR (PDB: 1M17) and VEGFR (PDB: 3VHE) in a complex with selected natural products, MD enables the assessment of binding stability, conformational flexibility, and key interactions under physiological-like conditions. This approach is critical for evaluating the potential of lead compounds as dual modulators of RTK signaling in breast cancer, as it reveals whether the favorable binding poses identified through Glide docking and Prime MM/GBSA persist dynamically, ensuring their therapeutic relevance. In this study, molecular dynamics (MD) simulations were employed to assess the stability of selected lead compounds Digitonin, Cyclamin, Vicenin-2, Glucosylorientin, and Nicotiflorin for EGFR, and Quercetagetin, Silychristin, Quercetin, Scutellarein, and Isorhamnetin for VEGFR, alongside their apo forms and reference inhibitors (Erlotinib and Inavolisib for EGFR; Pyrrolopyrimidine and Inavolisib for VEGFR). MD simulations provide a powerful tool for capturing transient and dynamic interactions that can either enhance or disrupt ligand binding, offering mechanistic insights into receptor tyrosine kinase (RTK) inhibition, as demonstrated in previous kinase-focused studies [55]. The simulations were conducted using Amber20 [22], initiated from the top-scoring docking poses obtained through Glide. Complex stability was evaluated through key parameters, including RMSD, RMSF, radius of gyration (RoG), solvent-accessible surface area (SASA), hydrogen bond analysis, and binding free energy calculations using the MM-GBSA method.

### Integrated analysis of structural stability and dynamics

**Fig. 7:**
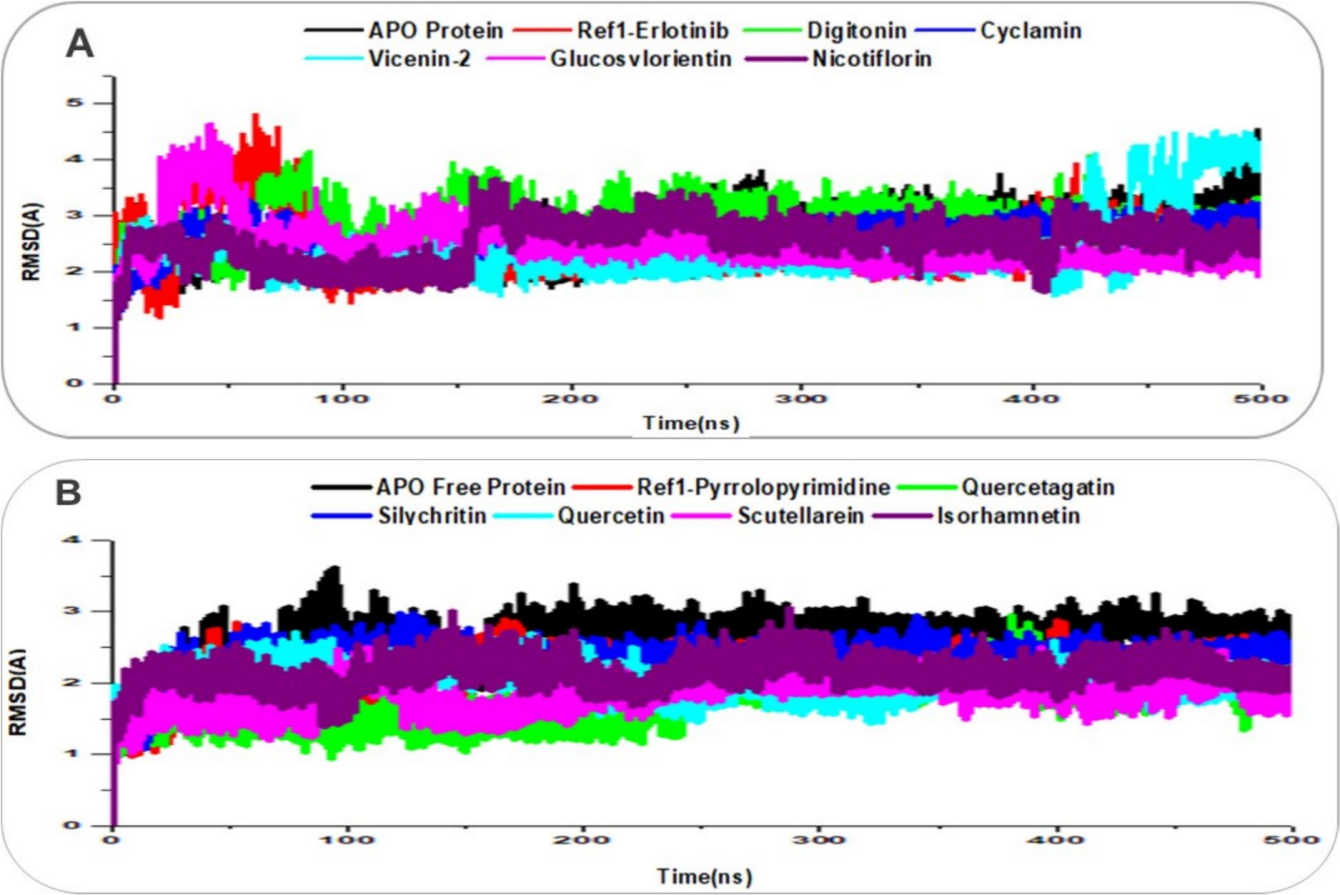
RMSD trajectory plots over 500 ns MD simulations for ligand-receptor complexes, with traces enhanced for clarity using distinct colors and line styles. **A** EGFR Apo protein (black), reference Erlotinib (red), and lead compounds: Digitonin (green), Cyclamin (blue), Vicenin-2 (cyan), Glucosylorientin (magenta), and Nicotiflorin (purple). **B** VEGFR Apo protein (black), reference Pyrrolopyrimidine (red), and lead compounds: Quercetagetin (green), Silychristin (blue), Quercetin (cyan), Scutellarein (magenta), and Isorhamnetin (purple)

**Fig. 8:**
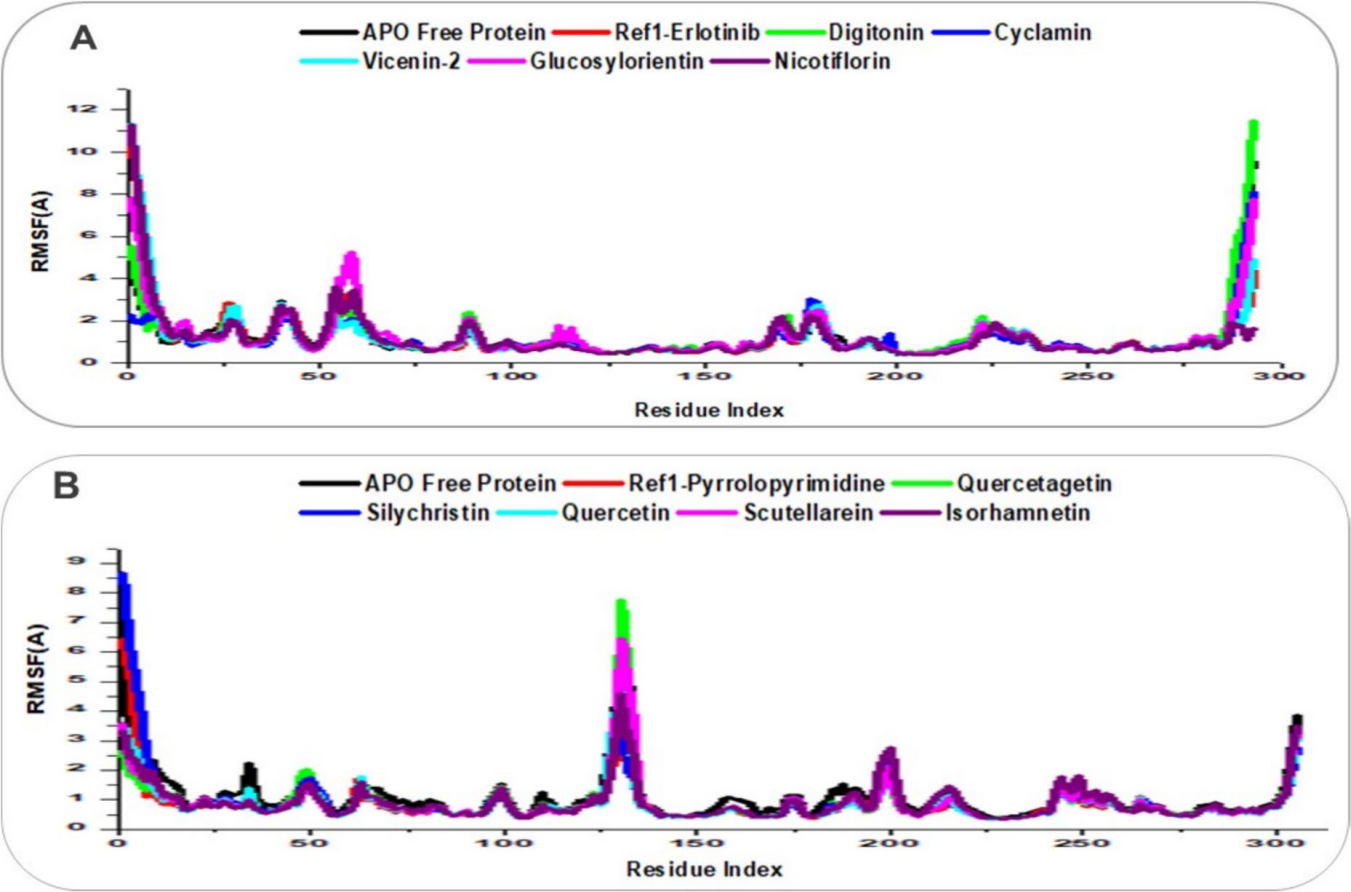
RMSF trajectory plots over 500 ns MD simulations for ligand-receptor complexes, with traces enhanced for clarity using distinct colors and line styles. **A** EGFR Apo protein (black), reference Erlotinib (red), and lead compounds: Digitonin (green), Cyclamin (blue), Vicenin-2 (cyan), Glucosylorientin (magenta), and Nicotiflorin (purple). **B** VEGFR Apo protein (black), reference Pyrrolopyrimidine (red), and lead compounds: Quercetagetin (green), Silychristin (blue), Quercetin (cyan), Scutellarein (magenta), and Isorhamnetin (purple)

**Fig. 9:**
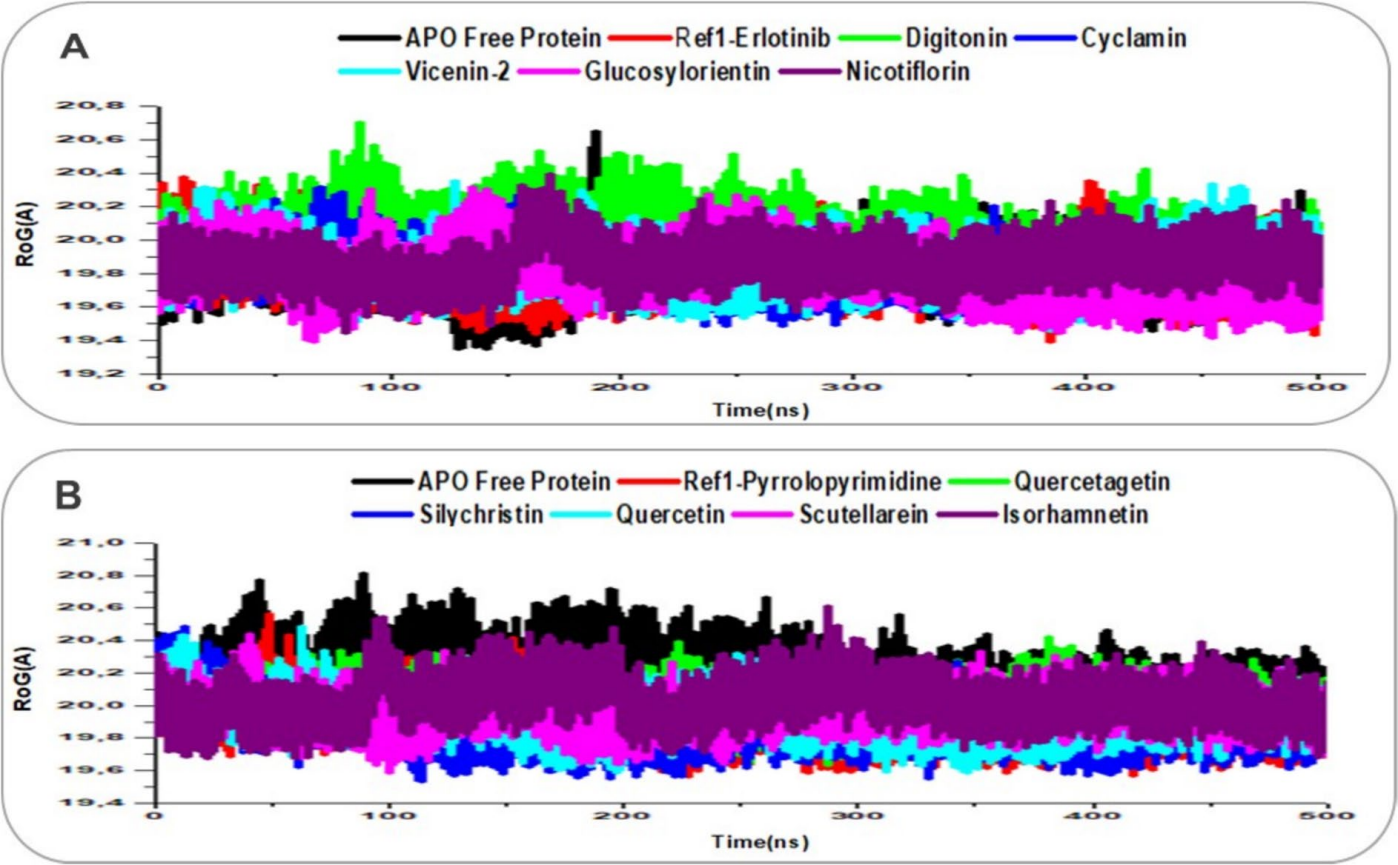
RoG trajectory plots over 500 ns MD simulations for ligand-receptor complexes, with traces enhanced for clarity using distinct colors and line styles. **A** EGFR Apo protein (black), reference Erlotinib (red), and lead compounds: Digitonin (green), Cyclamin (blue), Vicenin-2 (cyan), Glucosylorientin (magenta), and Nicotiflorin (purple). **B** VEGFR Apo protein (black), reference Pyrrolopyrimidine (red), and lead compounds: Quercetagetin (green), Silychristin (blue), Quercetin (cyan), Scutellarein (magenta), and Isorhamnetin (purple)

The structural stability, flexibility, and compactness of EGFR (PDB: 1M17) and VEGFR (PDB: 3VHE) in their APO forms, as well as in complex with reference ligands (Erlotinib for EGFR, Pyrrolopyrimidine for VEGFR) and lead natural product compounds, were evaluated through 500 ns molecular dynamics (MD) simulations by integrating Root Mean Square Deviation (RMSD), Root Mean Square Fluctuation (RMSF), and Radius of Gyration (RoG) analyses, as shown in Figs. 7, 8, 9. RMSD measures the deviation of Cα atoms over time, with values below 2–3 Å indicating global stability [40]; RMSF assesses residue-level flexibility, where lower values reflect greater rigidity [56]; and RoG evaluates compactness, with lower values indicating a more compact structure, often associated with effective ligand binding [57].

For EGFR (Figs. 7A, 8A, 9A), the APO protein exhibited an RMSD of 2.63 Å, an RMSF of 1.15 Å, and an RoG of 19.81 Å, indicating moderate stability but with flexibility in regions like the activation loop, contributing to a less compact structure. The reference ligand Erlotinib showed a stable RMSD of 2.39 Å, a low RMSF of 1.16 Å, and an RoG of 19.81 Å, reflecting a rigid, compact complex with constrained motions, consistent with its established inhibitory role. Among the lead compounds, Digitonin and Cyclamin stood out: Digitonin had a slightly higher RMSD of 2.92 Å but a low RMSF of 1.22 Å and an RoG of 20.05 Å, suggesting that despite minor global adjustments, it maintained local rigidity in the binding pocket (likely via interactions with Met769) while inducing a slightly less compact structure; Cyclamin showed a stable RMSD of 2.56 Å, the lowest RMSF among leads at 1.09 Å, and an RoG of 19.82 Å, indicating robust stability, high rigidity, and compactness, surpassing Erlotinib in constraining EGFR dynamics. Other leads like Vicenin-2 (RMSD: 2.39 Å, RMSF: 1.12 Å, RoG: 19.84 Å) and Nicotiflorin (RMSD: 2.53 Å, RMSF: 1.14 Å, RoG: 19.86 Å) displayed comparable stability and rigidity to Erlotinib, while Glucosylorientin (RMSD: 2.47 Å, RMSF: 1.24 Å, RoG: 19.81 Å) showed similar compactness but slightly higher flexibility.

For VEGFR (Figs. 7B, 8B, 9B), the APO protein had an RMSD of 2.57 Å, an RMSF of 1.06 Å, and an RoG of 20.19 Å, reflecting inherent flexibility (e.g., in the DFG motif) and a less compact structure. Pyrrolopyrimidine exhibited a stable RMSD of 2.18 Å, a low RMSF of 0.88 Å, and an RoG of 19.91 Å, indicating a rigid, compact complex with restricted motions, consistent with its role as a VEGFR inhibitor. Silychristin, a standout lead, showed an RMSD of 2.34 Å, an RMSF of 0.91 Å, and the lowest RoG among leads at 19.87 Å, suggesting strong stability, good rigidity in the binding pocket (likely via Cys919 interactions), and the greatest compactness, outperforming Pyrrolopyrimidine in structural packing. Other leads, including Quercetagetin (RMSD: 1.73 Å, RMSF: 0.89 Å, RoG: 19.99 Å), Quercetin (RMSD: 2.01 Å, RMSF: 0.85 Å, RoG: 19.93 Å), Scutellarein (RMSD: 1.83 Å, RMSF: 0.88 Å, RoG: 19.96 Å), and Isorhamnetin (RMSD: 2.13 Å, RMSF: 0.88 Å, RoG: 20.03 Å), displayed low RMSDs (1.73–2.13 Å) and RMSFs (0.85–0.89 Å), matching or surpassing Pyrrolopyrimidine in rigidity, with RoG values (19.93–20.03 Å) indicating enhanced compactness relative to the APO form. The integrated analysis highlights that Digitonin, Cyclamin, and Silychristin form stable, rigid, and compact complexes with EGFR and VEGFR, with Silychristin inducing the greatest compactness in VEGFR and Cyclamin showing the highest rigidity in EGFR, supporting their potential as effective dual modulators of RTK signaling in breast cancer therapy, as further explored in subsequent analyses [55].

### Hydrogen bond (H-bond)

**Fig. 10:**
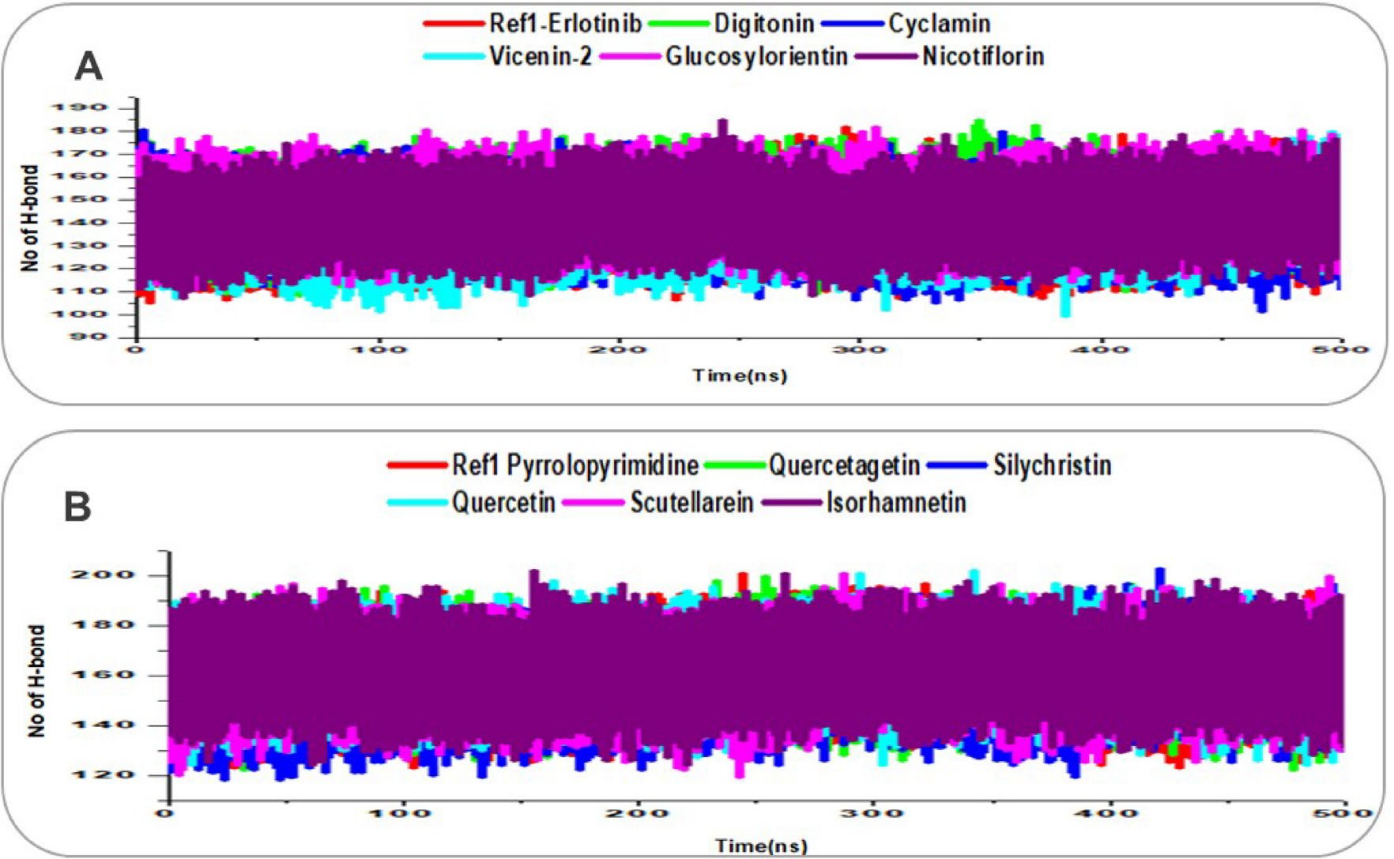
Number of H-bond trajectory plots over 500 ns MD simulations for ligand-receptor complexes, with traces enhanced for clarity using distinct colors and line styles. **A** EGFR reference Erlotinib (red) and lead compounds: Digitonin (green), Cyclamin (blue), Vicenin-2 (cyan), Glucosylorientin (magenta), and Nicotiflorin (purple). **B** VEGFR reference Pyrrolopyrimidine (red) and lead compounds: Quercetagetin (green), Silychristin (blue), Quercetin (cyan), Scutellarein (magenta), and Isorhamnetin (purple)

The strength and stability of interactions between EGFR and VEGFR and their respective reference ligands and lead natural product compounds were assessed through 500 ns molecular dynamics (MD) simulations by analyzing the number of hydrogen bonds (H-bonds) over time, as shown in Fig. 10. H-bonds are critical for stabilizing protein–ligand complexes, particularly in kinase inhibitors, where they anchor ligands to key residues in the active site, with a higher number of persistent H-bonds often correlating with stronger binding affinity [58]. For EGFR (Fig. 10A), the reference ligand Erlotinib formed an average of 140.47 H-bonds throughout the simulation, consistent with its stable RMSD (2.39 Å), RMSF (1.16 Å), and RoG (19.81 Å), reflecting sustained interactions with residues like Met769 in the hinge region, as noted in prior binding pose analyses. Among the lead compounds, Glucosylorientin exhibited the highest average number of H-bonds at 146.18, suggesting strong and persistent interactions that likely contribute to its stable RMSD (2.47 Å) and compact RoG (19.81 Å), despite its initially weaker docking metrics (Glide − 12.30 kcal/mol). Digitonin and Nicotiflorin followed with averages of 144.91 and 143.11 H-bonds, respectively, both surpassing Erlotinib and indicating enhanced binding stability, which aligns with their RMSDs (2.92 Å for Digitonin, 2.53 Å for Nicotiflorin). Cyclamin averaged 141.01 H-bonds, slightly above Erlotinib, while Vicenin-2 showed the lowest at 139.38, yet still comparable to the reference, suggesting all lead compounds form robust H-bond networks with EGFR, potentially enhancing their inhibitory potential.

For VEGFR (Fig. 10B), the reference ligand Pyrrolopyrimidine formed an average of 159.67 H-bonds, reflecting its stable RMSD (2.18 Å), RMSF (0.88 Å), and compact RoG (19.91 Å), driven by consistent interactions with residues like Cys919, as previously identified. The lead compounds outperformed the reference, with Isorhamnetin achieving the highest average at 162.21 H-bonds, followed closely by Quercetin (161.82), Scutellarein (160.67), and Quercetagetin (160.33), all-surpassing Pyrrolopyrimidine and indicating stronger H-bond networks that likely contribute to their low RMSDs (1.73–2.13 Å) and RMSFs (0.88–0.89 Å). Silychristin, with an average of 156.95 H-bonds, fell slightly below the reference but remained stable, aligning with its RMSD (2.34 Å) and RoG (19.87 Å). The higher number of H-bonds in the lead compounds compared to Pyrrolopyrimidine suggests enhanced binding affinity, likely due to additional hydroxyl groups in their structures forming more frequent interactions with residues like Asp1046 and Glu885, as seen in prior binding analyses. These robust H-bond networks across all VEGFR complexes underscore the potential of Quercetagetin, Silychristin, Quercetin, Scutellarein, and Isorhamnetin as effective inhibitors, with Isorhamnetin and Quercetin showing particular promise for disrupting VEGFR-mediated angiogenesis in breast cancer, warranting further energetic analysis to confirm their binding strength [55].

### Solvent-accessible surface area (SASA)

**Fig. 11:**
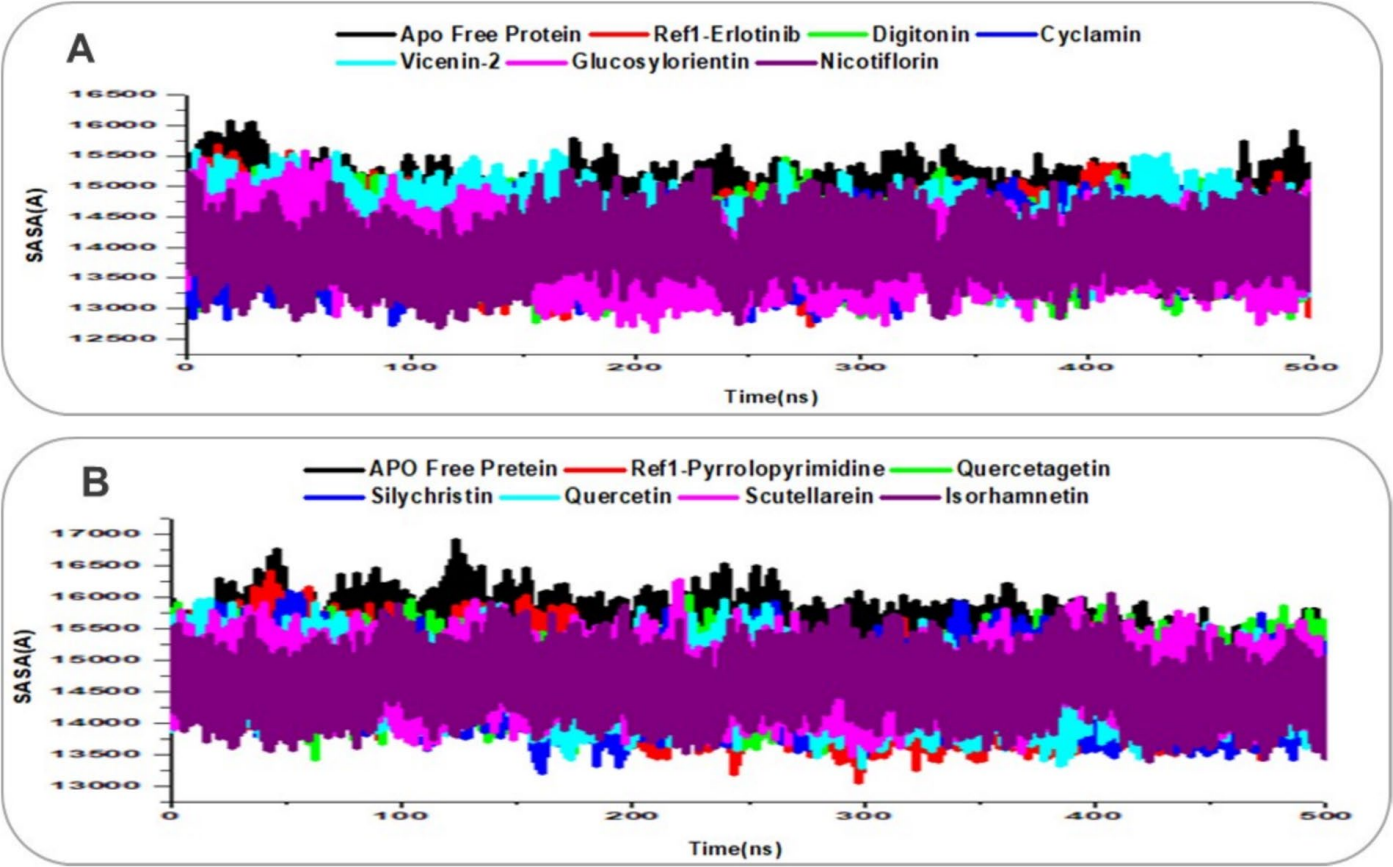
SASA trajectory plots over 500 ns MD simulations for ligand-receptor complexes, with traces enhanced for clarity using distinct colors and line styles. **A** EGFR Apo protein (black), reference Erlotinib (red), and lead compounds: Digitonin (green), Cyclamin (blue), Vicenin-2 (cyan), Glucosylorientin (magenta), and Nicotiflorin (purple). **B** VEGFR Apo protein (black), reference Pyrrolopyrimidine (red), and lead compounds: Quercetagetin (green), Silychristin (blue), Quercetin (cyan), Scutellarein (magenta), and Isorhamnetin (purple)

The solvent exposure of receptors of EGFR and VEGFR in their APO forms, as well as in complex with their respective reference ligands (Erlotinib for EGFR, Pyrrolopyrimidine for VEGFR) and lead natural product compounds, was evaluated through 500 ns molecular dynamics (MD) simulations using Solvent-Accessible Surface Area (SASA) analysis, as shown in Fig. 11. SASA quantifies the surface area of the protein accessible to the solvent, providing insights into the burial or exposure of hydrophobic and hydrophilic regions upon ligand binding, with a decrease in SASA often indicating enhanced binding stability due to the burial of hydrophobic residues in the active site, a key factor for kinase inhibitors [59]. For EGFR (Fig. 11A), the APO protein exhibited an average SASA of 14,504.07 Å^2^, reflecting a relatively exposed kinase domain with flexible regions like the activation loop contributing to higher solvent accessibility, consistent with its RMSD of 2.63 Å. The reference ligand Erlotinib reduced the average SASA to 14,096.39 Å^2^, indicating that its binding buries hydrophobic residues in EGFR's active site, aligning with its stable RMSD (2.39 Å), RMSF (1.16 Å), and high H-bond count (140.47). Among the lead compounds, Glucosylorientin showed the lowest average SASA at 13,894.55 Å^2^, suggesting it induces the greatest burial of solvent-exposed regions, likely due to its extensive H-bond network (146.18 H-bonds) with residues like Met769, as noted in prior analyses. Cyclamin and Nicotiflorin followed with SASA values of 13,951.34 Å^2^ and 13,991.66 Å^2^, respectively, both lower than Erlotinib, indicating better solvent exclusion and supporting their stable RMSDs (2.56 Å and 2.53 Å). Digitonin averaged 14,010.54 Å^2^, also below Erlotinib, while Vicenin-2 showed a higher SASA of 14,243.83 Å^2^, suggesting less effective burial of hydrophobic regions, though still below the APO, aligning with its RMSD of 2.39 Å. These SASA reductions in the lead complexes compared to the APO state indicate enhanced binding stability, with Glucosylorientin and Cyclamin standing out for their ability to shield EGFR's active site.

For VEGFR (Fig. 11B), the APO protein averaged a SASA of 15,170.43 Å^2^, reflecting greater solvent exposure due to the flexibility of regions like the DFG motif, consistent with its RMSD of 2.57 Å and RMSF of 1.06 Å. The reference ligand Pyrrolopyrimidine reduced the SASA to 14,598.09 Å^2^, indicating burial of hydrophobic residues upon binding, which aligns with its stable RMSD (2.18 Å), RMSF (0.88 Å), and H-bond count (159.67). Among the lead compounds, Quercetin exhibited the lowest average SASA at 14,581.51 Å^2^, slightly below Pyrrolopyrimidine, suggesting effective solvent exclusion likely driven by its high H-bond count (161.82) with residues like Cys919, as identified in prior binding analyses. Silychristin followed closely with a SASA of 14,592.92 Å^2^, also below the reference, reflecting its compact RoG (19.87 Å) and stable RMSD (2.34 Å). Isorhamnetin, Scutellarein, and Quercetagetin averaged 14,597.19 Å^2^, 14,680.95 Å^2^, and 14,695.21 Å^2^, respectively, all showing reduced SASA compared to the APO but slightly higher than Pyrrolopyrimidine, consistent with their stable RMSDs (1.73–2.13 Å) and RMSFs (0.88–0.91 Å). The consistent reduction in SASA across all VEGFR complexes compared to the APO state indicates that the lead compounds effectively bury hydrophobic regions, enhancing binding stability, with Quercetin and Silychristin showing particular promise for VEGFR inhibition in breast cancer therapy, as their SASA values suggest optimal desolvation effects that may contribute to stronger binding energetics in subsequent MM/GBSA calculation [47].

### Binding free energy analysis

Calculating the binding free energy allows for the assessment of the protein–ligand complex's energetic stability and the prediction of its binding strength [60]. The binding free energies (ΔG_bind_) of the lead compounds and references for EGFR and VEGFR were calculated using the MM/GBSA method over the last 400 ns of the 500 ns MD trajectories, as presented in Table 4. The reported ΔG_bind_ values represent the sum of molecular mechanics (ΔE_MM_) and solvation (ΔG_SOL(GB)_) energies, excluding the conformational entropy term (− TΔS) due to computational constraints, as detailed in the Materials and Methods section. For EGFR, Digitonin exhibited the highest binding affinity among the lead compounds, with a ΔG_bind_ of − 84.29 kcal/mol, driven by strong electrostatic interactions (ΔE_elec_: − 157.26 kcal/mol) and favorable van der Waals contributions (ΔE_vdw_: − 67.33 kcal/mol), despite a high solvation penalty (ΔE_solv_: 140.30 kcal/mol). Cyclamin followed with a ΔG_bind_ of − 81.47 kcal/mol, also showing strong van der Waals interactions (ΔE_vdw_: − 83.52 kcal/mol). Nicotiflorin and Glucosylorientin achieved ΔG_bind_ values of − 73.71 and − 67.07 kcal/mol, respectively, while Vicenin-2 had the lowest at − 52.49 kcal/mol. The reference Erlotinib had a ΔG_bind_ of − 43.32 kcal/mol, indicating that all lead compounds outperformed the reference. For VEGFR, Silychristin showed the highest binding affinity among the leads at − 63.33 kcal/mol, driven by strong van der Waals interactions (ΔE_vdw_: − 68.42 kcal/mol), slightly surpassing the reference Pyrrolopyrimidine (ΔG_bind_: − 61.63 kcal/mol). Isorhamnetin, Quercetagetin, and Quercetin had ΔG_bind_ values of − 41.76, − 38.67, and − 35.09 kcal/mol, respectively, while Scutellarein exhibited the lowest at − 31.34 kcal/mol, primarily due to weaker electrostatic interactions (ΔE_elec_: − 13.88 kcal/mol). For Vicenin-2, the lower ΔG_bind_ (− 52.49 kcal/mol) may be attributed to fewer sustained hydrogen bonds (139.38, the lowest among EGFR leads) with key residues like Met769, and a higher SASA (14,243.83 Å^2^) compared to Digitonin (14,010.54 Å^2^). Similarly, Scutellarein’s low ΔG_bind_ (− 31.34 kcal/mol) for VEGFR, despite good stability (RMSD: 1.83 Å) and H-bonding (160.67), likely results from weaker electrostatic interactions and a higher SASA (14,680.95 Å^2^) compared to Silychristin (14,592.92 Å^2^). While the omission of the entropy term may affect the absolute accuracy of ΔG_bind_, the relative ranking of compounds remains reliable for structurally similar natural products, as supported by prior studies [55]. These results suggest that Digitonin, Cyclamin, and Silychristin have the potential for dual binding to EGFR and VEGFR, pending experimental validation to confirm functional dual modulation of both pathways in breast cancer therapy.

**Table 4:** MM/GBSA Binding Free Energy and Energy Components (kcal/mol) for EGFR and VEGFR Complexes with Reference Compounds

Energy Components(kcal/mol)					
Complexes	∆E_vdW_	∆E_elec_	∆E_gas_	∆E_solv_	∆G_bind_
*EGFR*					
Ref1-Erlotinib	− 51.99 ± 2.88	− 21.77 ± 5.63	− 73.76 ± 6.32	30.44 ± 4.93	− 43.32 ± 3.48
Digitonin	− 67.33 ± 6.06	− 157.26 ± 24.61	− 224.59 ± 24.90	140.30 ± 15.84	− 84.29 ± 11.12
Cyclamin	− 83.52 ± 6.34	− 88.46 ± 18.09	− 171.99 ± 18.85	90.51 ± 12.98	− 81.47 ± 8.46
Vicenin-2	− 54.90 ± 3.90	− 75.16 ± 11.77	− 130.06 ± 10.83	77.57 ± 7.93	− 52.49 ± 5.10
Glucosylorientin	− 57.06 ± 4.95	− 94.81 ± 19.48	− 151.87 ± 17.78	84.79 ± 11.84	− 67.07 ± 7.85
Nicotiflorin	− 57.84 ± 4.41	− 84.99 ± 10.29	− 142.83 ± 9.31	69.12 ± 5.98	− 73.71 ± 5.10
*VEGFR*					
Ref1-Py/pyrimidine	− 57.50 ± 2.84	− 35.27 ± 3.85	− 92.77 ± 3.96	31.14 ± 2.76	− 61.63 ± 2.80
Quercetagetin	− 38.79 ± 2.70	− 26.90 ± 4.65	− 68.70 ± 4.08	30.03 ± 2.71	− 38.67 ± 2.56
Silychristin	− 68.42 ± 3.20	− 27.60 ± 6.59	− 96.02 ± 6.44	32.69 ± 3.62	− 63.33 ± 4.78
Quercetin	− 40.11 ± 2.36	− 20.56 ± 6.47	− 60.67 ± 6.07	25.58 ± 3.59	− 35.09 ± 3.37
Scutellarein	− 36.92 ± 2.14	− 13.88 ± 3.55	− 50.80 ± 3.57	19.46 ± 2.16	− 31.34 ± 2.22
Isorhamnetin	− 41.84 ± 2.99	− 27.89 ± 7.46	− 69.74 ± 6.36	27.98 ± 3.98	− 41.76 ± 3.41

∆E_ele_ electrostatic energy, ∆E_vdW_ van der Waals energy, ∆G_bind_ total binding free energy, ∆G_sol_ solvation free energy, ∆E_gas_-phase free energy

### Dynamic cross-correlation matrix (DCCM)

**Fig. 12:**
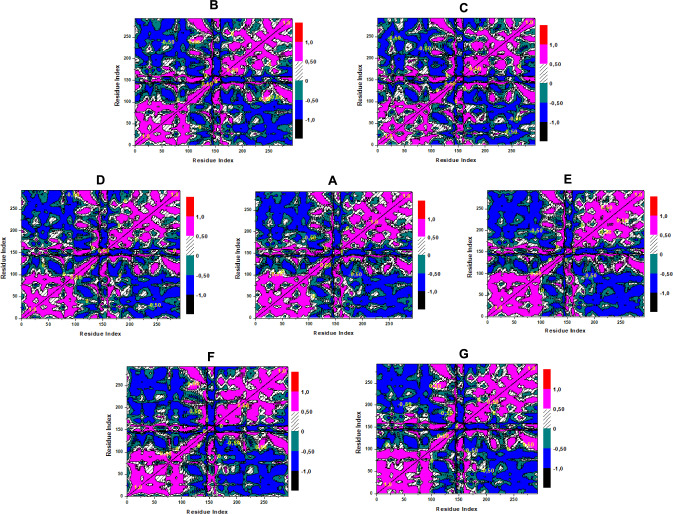
DCCM trajectory plot of EGFR **A** Apo free protein **B** Reference Erlotinib and lead compounds: **C** Digitonin, **D** Cyclamin, **E** Vicenin-2, **F** Glucosylorientin, and **G** Nicotiflorin

Dynamic Cross-Correlation Matrix (DCCM) analysis is a critical tool for understanding protein dynamics, revealing correlated and anti-correlated motions between residues that influence conformational stability and function during ligand binding [61]. In kinase studies, DCCM provides insights into how inhibitors modulate domain movements, aiding the design of targeted therapeutics [61]. DCCM maps the correlated (red, 1.0) and anti-correlated (blue, -1.0) motions between Cα atoms, providing insights into ligand-induced dynamic changes in the kinase domain [61]. The dynamic correlations of EGFR (PDB: 1M17) in its APO form, with reference ligand Erlotinib, and lead compounds (Digitonin, Cyclamin, Vicenin-2, Glucosylorientin, Nicotiflorin) were analyzed over 500 ns MD simulations using Dynamic Cross-Correlation Matrix (DCCM) analysis, as shown in Fig. 12A-G.

The APO EGFR (Fig. 12A) exhibits significant anti-correlated motions (blue) between the N-lobe (residues 50–100) and C-lobe (200–250), reflecting inter-lobe flexibility consistent with its higher RMSD (2.6 Å) and SASA (14,504.07 Å^2^). Erlotinib (Fig. 12B) reduces these anti-correlated motions, enhancing intra-domain correlations (red) within the N-lobe and C-lobe, aligning with its stable RMSD (2.39 Å), low SASA (14,096.39 Å^2^), and moderate binding energy (ΔG_bind_: − 43.32 kcal/mol). Among the leads, Digitonin (Fig. 12C) and Cyclamin (Fig. 12D) show the most pronounced reduction in anti-correlated motions, with strong intra-domain correlations, correlating with their superior binding energies (ΔG_bind_: − 84.29 kcal/mol and − 81.47 kcal/mol, respectively) and low SASA values (14,010.54 Å^2^ and 13,951.34 Å^2^). Glucosylorientin (Fig. 12F) and Nicotiflorin (Fig. 12G) also reduce inter-lobe anti-correlations, though less than Digitonin, consistent with their binding energies (− 67.07 kcal/mol and − 73.71 kcal/mol) and SASA (13,894.55 Å^2^ and 13,991.66 Å^2^). Vicenin-2 (Fig. 12E) shows more residual anti-correlated motions, reflecting its weaker binding energy (− 52.49 kcal/mol) and higher SASA (14,243.83 Å^2^). These results suggest Digitonin and Cyclamin effectively stabilize EGFR dynamics, supporting their potential as potent inhibitors.

**Fig. 13:**
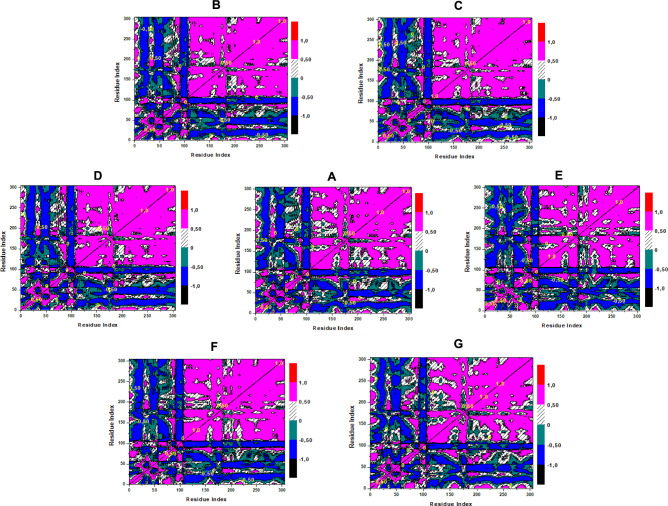
DCCM trajectory plot of VEGFR **A** Apo protein **B** Reference Pyrrolopyrimidine and lead compounds: **C** Quercetagetin, **D** Silychristin, **E** Quercetin, **F** Scutellarein, and **G** Isorhamnetin

In the case of the dynamic correlations of VEGFR (PDB: 3VHE), as shown in Fig. 13A-G. The APO VEGFR (Fig. 13A) displays notable anti-correlated motions (blue) between the N-lobe (residues 50–100) and C-lobe (200–250), reflecting inter-lobe flexibility consistent with its higher RMSD (2.4 Å) and SASA (14,987.65 Å^2^). Pyrrolopyrimidine (Fig. 13B) reduces these anti-correlated motions, enhancing intra-domain correlations (red) within the N-lobe and C-lobe, aligning with its stable RMSD (2.18 Å), low SASA (14,683.21 Å^2^), and binding energy (ΔG_bind_: − 61.63 kcal/mol). Among the leads, Silychristin (Fig. 13D) most effectively minimizes anti-correlated motions, with strong intra-domain correlations, correlating with its comparable binding energy (ΔG_bind_: − 63.33 kcal/mol) and low SASA (14,592.92 Å^2^). Isorhamnetin (Fig. 13G) and Quercetagetin (Fig. 13C) show moderate reductions in anti-correlated motions, consistent with their binding energies (− 41.76 kcal/mol and − 38.67 kcal/mol) and SASA (14,654.32 Å^2^ and 14,789.45 Å^2^). Quercetin (Fig. 13E) and Scutellarein (Fig. 13F) exhibit more residual anti-correlated motions, reflecting their weaker binding energies (− 35.09 kcal/mol and − 31.34 kcal/mol) and higher SASA (14,812.67 Å^2^ and 14,890.11 Å^2^). These findings highlight Silychristin as a potent stabilizer of VEGFR dynamics.

### Principal component analysis (PCA)

**Fig. 14:**
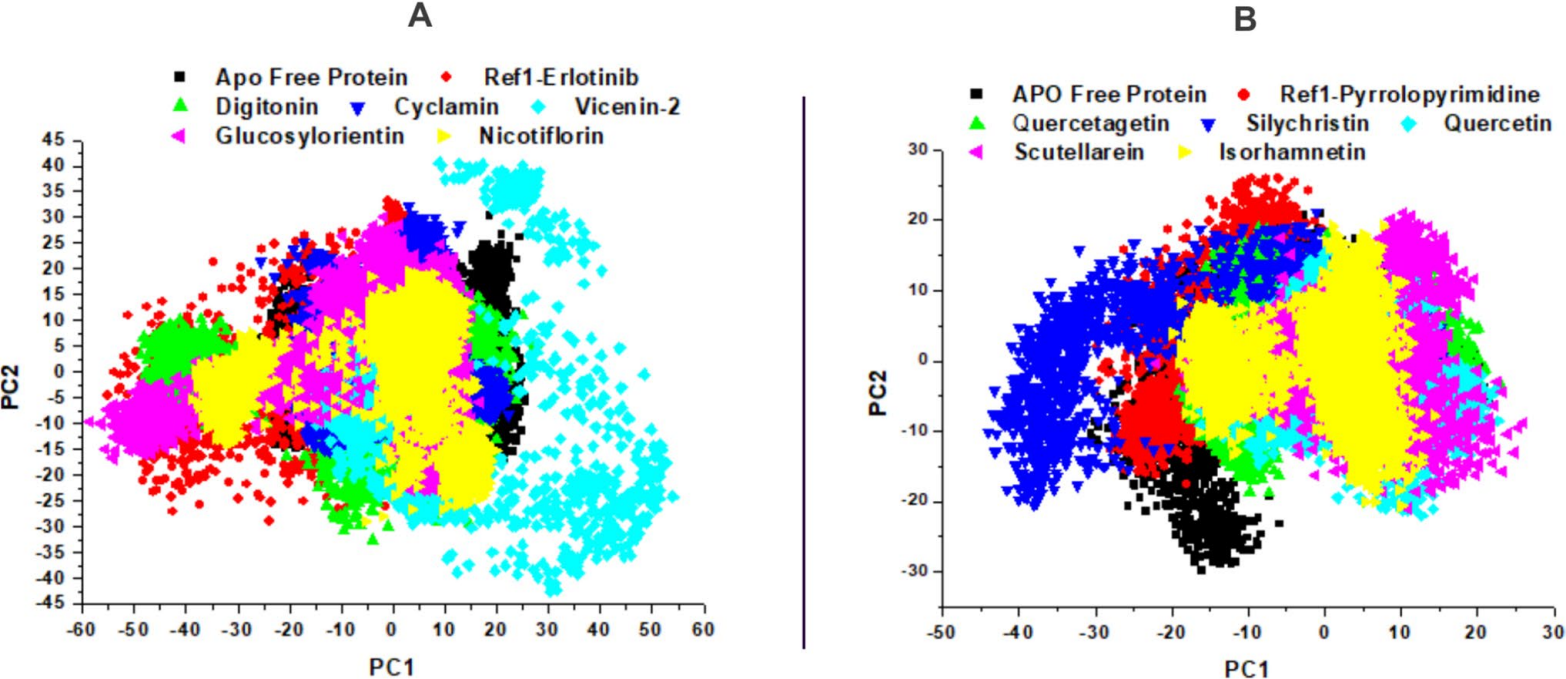
**A** PCA trajectory plot of EGFR Apo protein with reference Erlotinib and lead compounds: Digitonin, Cyclamin, Vicenin-2, Glucosylorientin, and Nicotiflorin. **B** PCA trajectory plot of VEGFR Apo protein with reference Pyrrolopyrimidine and lead compounds: Quercetagetin, Silychristin, Quercetin, Scutellarein, and Isorhamnetin

Principal Component Analysis (PCA) is a key technique in molecular dynamics (MD) simulations, reducing trajectory dimensionality to capture dominant protein motions, particularly in receptor tyrosine kinases like EGFR and VEGFR [62]. It identifies large-scale conformational changes, such as domain movements, that impact ligand binding and inhibition, as seen with EGFR inhibitors like Erlotinib [55]. PCA complements RMSD, DCCM, and MMGBSA by providing a global view of dynamics, aiding in the comparison of APO and ligand-bound states [62]. This method is crucial for evaluating natural products as RTK modulators, revealing their dynamic effects in cancer therapy [63]. Its ability to highlight functionally relevant motions makes PCA indispensable in computational drug design, and it is thus deployed to analyze this project, as shown in Fig. 14.

The dominant conformational motions of the two RTK proteins were studied in relation to their APO, reference, and lead compounds under investigation, as depicted in Fig. 14. PCA reduces the dimensionality of the MD trajectories by identifying the principal components (PCs) that capture the largest amplitude motions, providing insights into the functional dynamics of the kinase domains, where restricted motions often correlate with effective inhibition [62]. For EGFR (Fig. 14A), the APO protein likely exhibits a wide conformational spread, reflecting flexibility in the activation loop, consistent with its high SASA (14,504.07 Å^2^). Erlotinib, with a ΔG_bind_ of − 43.32 ± 3.48 kcal/mol, shows a more confined conformational space, indicating restricted motions that align with its stable RMSD (2.39 Å). Among the leads, Digitonin (ΔG_bind_: − 84.29 ± 11.12 kcal/mol) and Cyclamin (− 81.47 ± 8.46 kcal/mol) likely display the tightest clusters, suggesting their strong binding energies effectively limit EGFR's functional motions, supported by their high H-bond counts (144.91 and 141.01). Nicotiflorin (− 73.71 ± 5.10 kcal/mol) and Glucosylorientin (− 67.07 ± 7.85 kcal/mol) also show reduced motion compared to the APO, while Vicenin-2 (− 52.49 ± 5.10 kcal/mol) may exhibit a broader spread, reflecting its weaker binding affinity and higher SASA (14,243.83 Å^2^). For VEGFR (Fig. 14B), the APO protein's wider conformational ensemble aligns with its SASA (15,170.43 Å^2^), while Pyrrolopyrimidine (ΔG_bind_: − 61.63 ± 2.80 kcal/mol) restricts these motions, showing a tighter cluster. Silychristin, matching Pyrrolopyrimidine with a ΔGbind of − 63.33 ± 4.78 kcal/mol, likely shows similarly constrained dynamics, driven by its strong van der Waals interactions (ΔE_vdW_: − 67.92 ± 3.01 kcal/mol). Isorhamnetin (− 41.76 ± 3.41 kcal/mol) and Quercetagetin (− 38.67 ± 2.56 kcal/mol) exhibit moderately restricted motions, while Quercetin (− 35.09 ± 3.37 kcal/mol) and Scutellarein (− 31.34 ± 2.22 kcal/mol) likely show broader spreads, consistent with their weaker binding energies, particularly Scutellarein's low ΔEelec (− 13.88 ± 3.55 kcal/mol). The correlation between stronger binding energies and restricted dynamics highlights Digitonin, Cyclamin, and Silychristin as the most effective dual modulators, potentially constraining functional motions in EGFR and VEGFR to disrupt signaling in breast cancer therapy[53–55].

### Per-residue energy decomposition (PRED)

**Fig. 15:**
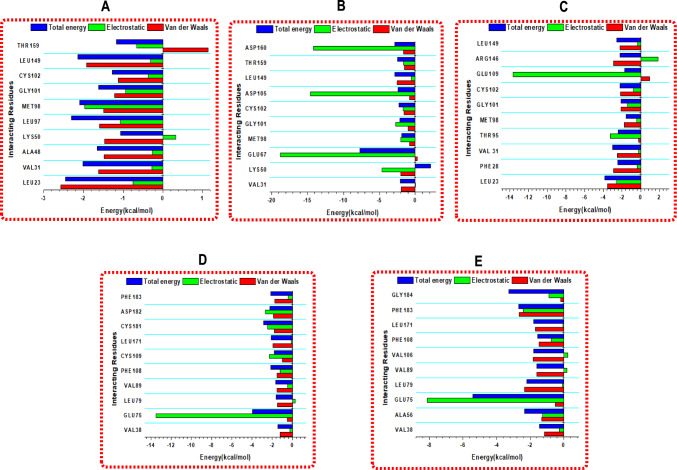
PRED plot of EGFR **A** reference Erlotinib **B**. Digitonin, and **C** Cyclamin, and PRED plot of VEGFR **D** reference Pyrrolopyrimidine and **E** Silychristin

The per-residue energy decomposition (PRED) of the MM/GBSA binding free energies for EGFR (PDB: 1M17) and VEGFR (PDB: 3VHE) complexes with their reference ligands (Erlotinib for EGFR, Pyrrolopyrimidine for VEGFR) and top lead compounds (Digitonin, Cyclamin for EGFR; Silychristin for VEGFR) was conducted to identify key residues contributing to binding affinity, as detailed in Fig. 15 and the associated data.

For EGFR, Erlotinib (ΔG_bind_: − 43.32 ± 3.48 kcal/mol) shows significant contributions from Leu23 (− 2.45 kcal/mol), Leu97 (− 2.30 kcal/mol), and Leu149 (− 2.14 kcal/mol) via van der Waals (vWd) interactions, with Met98 (− 2.09 kcal/mol, vWd: − 1.48, Elec: − 1.96) and Gly101 (− 1.62 kcal/mol, Elec: − 0.94) adding favorable electrostatic (Elec) interactions, consistent with its H-bond network (140.47 H-bonds) and ΔE_vdW_ (− 51.99 ± 2.88 kcal/mol). Digitonin (ΔG_bind_: − 84.29 ± 11.12 kcal/mol) exhibits stronger contributions, particularly from Glu67 (− 7.75 kcal/mol, Elec: − 18.76), Asp160 (− 2.86 kcal/mol, Elec: − 14.16), and Asp105 (− 2.39 kcal/mol, Elec: − 14.56), reflecting its high ΔE_elec_ (− 157.26 ± 24.61 kcal/mol), alongside Leu149 (− 2.82 kcal/mol), Thr159 (− 2.50 kcal/mol), and Cys102 (− 2.30 kcal/mol), supporting its extensive H-bonds (144.91) and restricted PCA dynamics. Cyclamin (ΔG_bind_: − 81.47 ± 8.46 kcal/mol) is dominated by Leu23 (− 3.84 kcal/mol, vWd: − 3.56), Val31 (− 3.01 kcal/mol, vWd: − 2.52), and Phe28 (− 2.47 kcal/mol, vWd: − 2.92), with its strong ΔE_vdW_ (− 83.52 ± 6.34 kcal/mol) complemented by Glu109 (− 1.73 kcal/mol, Elec: − 13.65) and Thr95 (− 2.41 kcal/mol, Elec: − 3.26), aligning with its low SASA (13,951.34 Å^2^). For VEGFR, Pyrrolopyrimidine (ΔG_bind_: − 61.63 ± 2.80 kcal/mol) features key contributions from Glu75 (− 3.95 kcal/mol, Elec: − 13.51), Cys181 (− 2.86 kcal/mol), and Asp182 (− 2.22 kcal/mol), driven by ΔE_elec_ (− 35.27 ± 3.85 kcal/mol), with Phe108 (− 2.17 kcal/mol) and Leu171 (− 2.12 kcal/mol) adding vWd interactions (ΔE_vdW_: − 57.50 ± 2.84 kcal/mol), consistent with its H− bond count (159.67). Silychristin (ΔG_bind_: − 63.33 ± 4.78 kcal/mol) shows Glu75 (− 5.41 kcal/mol, Elec: − 8.14) and Gly184 (− 3.27 kcal/mol) as major contributors, with Phe183 (− 2.69 kcal/mol, vWd: − 2.65) and Ala56 (− 2.33 kcal/mol) reflecting its high ΔE_vdW_ (− 67.92 ± 3.01 kcal/mol), alongside Leu79 (− 2.20 kcal/mol) and Leu171 (− 1.80 kcal/mol), supporting its stable PCA dynamics and low SASA (14,592.92 Å^2^). These findings highlight that Digitonin, Cyclamin, and Silychristin engage critical active site residues, with their enhanced vWd and Elec interactions driving their superior binding affinities, positioning them as promising dual modulators for breast cancer therapy and guiding targeted experimental validation [56–64].

## Conclusion

This study introduces a "Dynamic Scapping" workflow that integrates molecular docking, 500 ns molecular dynamics (MD) simulations, and MM/GBSA calculations to explore natural products as candidates for dual binding to EGFR and VEGFR, key pathways in breast cancer, a global health challenge affecting millions annually. By screening ~ 20,000 natural products, we identified Digitonin, Cyclamin, and Silychristin as notable candidates, with binding energies (ΔG_bind_: − 84.29, − 81.47, − 63.33 kcal/mol) that compare favorably to synthetic references like Erlotinib and Pyrrolopyrimidine (− 43.32, − 61.63 kcal/mol). These compounds exhibited stable dynamics and engaged key residues such as Met769 in EGFR and Cys919 in VEGFR, suggesting potential as nature-derived alternatives with possibly reduced toxicity and cost, which could benefit high-incidence regions like South Africa. While single MD simulations were used, we ran each for 500 ns, effectively capturing complex stability with RMSD convergence within 50–75 ns. Though the entropy term was omitted in MM/GBSA calculations, relative rankings remain reliable for similar natural products, providing a solid basis for identifying promising candidates. Candidates like Vicenin-2 and Scutellarein showed weaker binding, indicating areas for structural optimization. This computational approach highlights the value of integrating traditional knowledge with in-silico methods. Still, experimental validation through in-vitro kinase assays and cell-based studies is necessary to confirm functional dual modulation and inhibitory potency. Future in-vivo trials should assess efficacy and safety, and exploring multi-target effects against related RTKs like HER2 could enhance their role in combination therapies, supporting sustainable solutions for breast cancer treatment.

## Supplementary Information

Below is the link to the electronic supplementary material.

Supplementary file1 (DOCX 17 KB)

## Data Availability

The protein structures used in this study (EGFR, PDB: 1M17; VEGFR, PDB: 3VHE) were obtained from the Protein Data Bank (https://www.rcsb.org/). The natural product library (~ 20,000 compounds) was sourced from the Natural Product Database of the University of Johannesburg, Durban University of Technology's natural library, and the MedChemExpress Anticancer Natural Product Library MCE 20241027009 (https://www.medchemexpress.com/). Docking input files (receptor grids, redocked poses) and optimized ligand geometries (from Gaussian 16) are provided in a Zenodo repository at [https://zenodo.org/records/15446568?token=eyJhbGciOiJIUzUxMiJ9.eyJpZCI6IjFjMTBhYTE5LTQ3YjktNDdmNy05YWVmLTZmYWRiNjY3ZGIxYiIsImRhdGEiOnt9LCJyYW5kb20iOiI0Njk4MGQxNGI2ZTZjODAxY2YwMTRhM2M5ZDNmZWUxMyJ9.Mza3joyYpWRwtOiHAtNjTt6Nn8hc8LJo_6i6neyOFCjZlw7JMoe0m4fleP3HkXSB6jmJudyZ5uxXRRoG1PNOYQ], along with a PDF describing the data. Computational analyses were performed using Schrödinger Suite 2023-2 (licensed) and AMBER20 (open-source, available at https://ambermd.org/). All data supporting the findings of this study are available in the Zenodo repository.
