# Supplementary material for: A dynamic scapping workflow for RTK domains: computational modeling of natural products as dual modulators of EGFR and VEGFR signaling in breast cancer
Source: Mol Divers. 2025 Jul 10;30(1):1459–85. doi: 10.1007/s11030-025-11263-x (PMC12926249; doi:10.1007/s11030-025-11263-x)
Supplement: Supplementary file 1 — Supplementary file1 (DOCX 17 KB) [file 11030_2025_11263_MOESM1_ESM.docx]

**Supporting Information**

**Dynamic Scapping of RTK Domains: Computational Modeling of Natural Products as Dual Modulators of EGFR and VEGFR Signaling in Breast Cancer**

Vincent A. Obakachi^1*^, Krishna K. Govender^1*^, Penny P. Govender^1^

^1^Department of Chemical Sciences,

University of Johannesburg,

Doornfontein Campus, P.O. Box 17011,

Johannesburg 2028, South Africa.

*Corresponding authors:

krishnag@uj.ac.za, vincentobakachi@gmail.com

- **Natural Product Library:** The library of ~20,000 natural product compounds was sourced from the Natural Product Database of the University of Johannesburg, Durban University of Technology's natural library, and the MedChemExpress Anticancer Natural Product Library. Structures are available in "natural_products_library.sdf" (SDF Format).
- **Docking Input Files:** Receptor grid files for EGFR (PDB: 1M17) and VEGFR (PDB: 3VHE) are available in "egfr_grid.zip" and "vegfr_grid.zip". Redocked poses (post-DFT optimization) for lead compounds and references are in "docking_poses.zip" (PDB format).
- **Optimized Ligand Geometries**: DFT-optimized geometries (B3LYP/def2-TZVP) of lead compounds and references are provided in "optimized_ligands.zip" (MOL2 format).
- The Supporting Information data files are hosted on Zenodo with a private link for reviewers:[https://zenodo.org/records/15446568?token=eyJhbGciOiJIUzUxMiJ9.eyJpZCI6IjFjMTBhYTE5LTQ3YjktNDdmNy05YWVmLTZmYWRiNjY3ZGIxYiIsImRhdGEiOnt9LCJyYW5kb20iOiI0Njk4MGQxNGI2ZTZjODAxY2YwMTRhM2M5ZDNmZWUxMyJ9.Mza3joyYpWRwtOiHAtNjTt6Nn8hc8LJo_6i6neyOFCjZlw7JMoe0m4fleP3HkXSB6jmJudyZ5uxXRRoG1PNOYQ]
